# The Bioactive Compounds of *Epimedium* and Their Potential Mechanism of Action in Treating Osteoporosis: A Network Pharmacology and Experimental Validation Study

**DOI:** 10.3390/ph17060706

**Published:** 2024-05-29

**Authors:** Huizhong Dong, Fen Tang, Zilu Zhao, Wenxuan Huang, Xiangyang Wan, Zhanying Hong, Ying Liu, Xin Dong, Si Chen

**Affiliations:** 1 School of Medicine, Shanghai University, Shanghai 200444, China; 2 School of Pharmacy, Naval Medical University, 325 Guohe Road, Shanghai 200433, China; 3 Institute of Translational Medicine, Shanghai University, 99 Shangda Road, Shanghai 200444, China; liuchanger1984@163.com

**Keywords:** osteoporosis, *Epimedium*, 2″-O-RhamnosylIcariside II, network pharmacology, HIF-1α

## Abstract

Osteoporosis is a global health challenge characterized by bone loss and microstructure deterioration, which urgently requires the development of safer and more effective treatments due to the significant adverse effects and limitations of existing drugs for long-term treatment. Traditional Chinese medicine, like *Epimedium*, offers fewer side effects and has been used to treat osteoporosis, yet its active compounds and pharmacological mechanisms remain unclear. In this study, 65 potential active compounds, 258 potential target proteins, and 488 pathways of *Epimedium* were identified through network pharmacology analysis. Further network analysis and review of the literature identified six potential active compounds and HIF-1α for subsequent experimental validation. In vitro experiments confirmed that 2″-O-RhamnosylIcariside II is the most effective compound among the six potential active compounds. It can promote osteoblast differentiation, bind with HIF-1α, and inhibit both HIF-1α gene and protein expression, as well as enhance COL1A1 protein expression under hypoxic conditions. In vivo experiments demonstrated its ability to improve bone microstructures and reduce bone loss by decreasing bone marrow adipose tissue, enhancing bone formation, and suppressing HIF-1α protein expression. This study is the first to describe the therapeutic effects of 2-O-RhamnosylIcariside II on osteoporosis, which was done, specifically, through a mechanism that targets and inhibits HIF-1α. This study provides a scientific basis for the clinical application of *Epimedium* and offers a new candidate drug for the treatment of osteoporosis. Additionally, it provides new evidence supporting HIF-1α as a therapeutic target for osteoporosis.

## 1. Introduction

Osteoporosis is a systemic skeletal disorder characterized by decreased bone mass and the deterioration of bone microstructure, resulting in increased bone fragility and a greater risk of fractures [1]. Although osteoporosis patients usually have no symptoms, a certain number of patients experience fractures as a result [2]. The lack of estrogen in postmenopausal women significantly increases their vulnerability to osteoporosis, given estrogen’s vital function in maintaining bone quality and quantity [3,4]. Estrogen deficiency accelerates bone loss and structural deterioration by promoting bone resorption and inhibiting bone formation. This process results in the disruption and thinning of trabecular bone, thereby significantly elevating the fracture risk [5,6]. Currently, the FDA (Food and Drug Administration) has approved several drugs for osteoporosis treatment, including selective estrogen receptor modulators, bisphosphonates, RANKL inhibitors, and teriparatide [7]. While these drugs can effectively improve related indicators and prevent fractures, their potential for significant side effects from long-term use brings additional physical and economical challenges for patients, such as esophagitis, osteonecrosis of the jaw, and hypercalcemia [8,9]. Therefore, it is crucial to develop new osteoporosis treatment strategies that are effective and have fewer side effects.

Traditional Chinese medicine, abundant in natural compounds, has garnered a wealth of experience over thousands of years and is generally associated with fewer adverse effects from long-term use compared to certain chemically synthesized medications [10,11,12,13,14]. *Epimedium* is a traditional Chinese herbal medicine that nourishes kidney yang, strengthens muscles and bones, and eliminates rheumatism [15]. Currently, over 260 compounds have been identified in *Epimedium*, with Icariin, its representative active ingredient, being extensively documented for its anti-osteoporosis effects through multiple signaling pathways [16,17,18]. For instance, Xu et al. [19] reported Icariin’s role in promoting osteocalcin transcription through STAT3 activation, thereby facilitating osteoblast differentiation in mBMSCs. Similarly, Zhou et al. [20] demonstrated that Icariin acts as a phytoestrogen, activating ERα and AKT phosphorylation by inducing the IGF-1 signaling pathway in MC3T3-E1 cells, and thus offering a potential treatment for postmenopausal osteoporosis. Although *Epimedium* has been used to treat osteoporosis for thousands of years, only 36 compounds in *Epimedium* have been reported to exhibit anti-osteoporosis effects in vitro or in vivo. Except for Icariin, investigation into how other active components in *Epimedium* treat osteoporosis is notably lacking. Thus, further research is urgently needed to elucidate the active components and mechanisms underlying the anti-osteoporosis effects of *Epimedium*.

Network pharmacology is an innovative approach that explores the complex interactions between drugs, proteins, and diseases through the lens of network science to understand the holistic effects of medications on biological systems. It holds significant potential for unraveling the multifaceted therapeutic mechanisms of traditional Chinese medicine, enabling a deeper understanding of its synergistic effects on multiple biological targets [21]. In this study, we performed network pharmacology analysis to screen potential active compounds in *Epimedium* and predict their potential action mechanisms in treating osteoporosis. Both in vitro and in vivo experiments were conducted to validate the results of our network pharmacology analysis. The findings will provide strong scientific support for *Epimedium*’s clinical use in osteoporosis treatment, and introduce a new candidate for the treatment of osteoporosis.

## 2. Results

### 2.1. Identification of Potential Anti-Osteoporosis Compounds from Epimedium and Pathway Enrichment by Network Pharmacology Analysis

#### 2.1.1. Databases of *Epimedium* Chemical Components and Known Anti-Osteoporosis Compounds

A total of 116 of *Epimedium*’s chemical compounds were identified through searches in databases, the literature, and books. These include 86 flavonoids, 7 acids, 5 esters, 3 glycosides, 3 hydrocarbons, 3 lignans, 3 phenols, 2 steroids, and 4 other types of compounds (Supplementary Table S1). Among these, 36 compounds have been identified with reported anti-osteoporosis activities either in vitro or in vivo (Supplementary Table S2). Notably, 15 active compounds, including Kaempferol, Icariside II, Icariin, Linoleic Acid, Epimedin B, Tricin, Sagittatoside B, Sagittatoside A, p-Coumaric Acid, Inositol, Icaritin, Hyperoside, Epimedin C, Anhydroicaritin, and Icariside B2 have been specifically reported to exert anti-osteoporosis effects through specific pathways and mechanisms.

#### 2.1.2. Potentially Active Compounds Prediction through the Construction of a Compound-Compound Interaction Network

**Figure 1:**
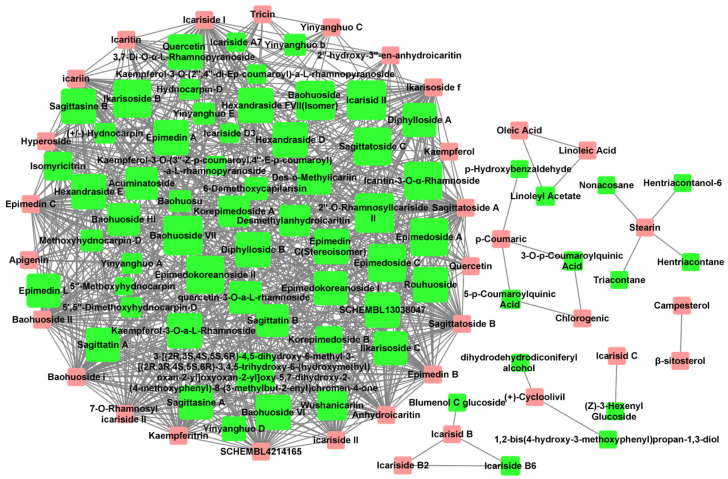
Potential active compound–compound interaction network in *Epimedium*. The pink nodes represent compounds explicitly reported to have anti-osteoporosis effects in vitro or in vivo. The green nodes represent potentially active compounds that share a structural similarity greater than 0.4 with the red nodes. The node size for potential active compounds (green nodes) was positively related to T_c_. Interactions between compounds are depicted by lines connecting the nodes.

The Tanimoto coefficient of similarity (T_c_) was calculated between 36 *Epimedium* compounds known for their anti-osteoporosis effects and another 80 compounds that had not been previously reported in the literature to possess such activity. Compounds with an average T_c_ greater than 0.4 were suggested to have potential anti-osteoporosis activity, resulting in a total of 731 compound–compound interaction pairs (Supplementary Table S3). A potential active compound–compound interaction network was constructed based on these pairs (Figure 1), with 33 compounds (pink nodes) known for their anti-osteoporosis effects that were mainly at the edge of the network, and 65 potentially active compounds (green nodes) that were mainly at the center of the network. Among the 65 potentially active compounds, approximately 75% are flavonoids, with the rest comprising acids, lignans, sterols, and other types of compounds.

#### 2.1.3. Selection of Potential Active Compounds for Experimental Validation

To discover new and unreported active compounds for osteoporosis treatment, we focused here on selecting candidates from a group of 65 potentially active compounds for experimental validation. These candidates were identified through network analysis for their structural similarities to known anti-osteoporosis compounds. Six potential active compounds were finally selected for experimental validation based on two key criteria: average T_c_ similarity and commercial availability (Table 1). This dual-criterion approach ensured that the selected compounds not only showed a high probability of biological activity due to their structural resemblance to effective compounds but were also immediately accessible for experimental testing.

#### 2.1.4. Selection of HIF-1α as Potential Target Protein for Further Experimental Validation

**Figure 2:**
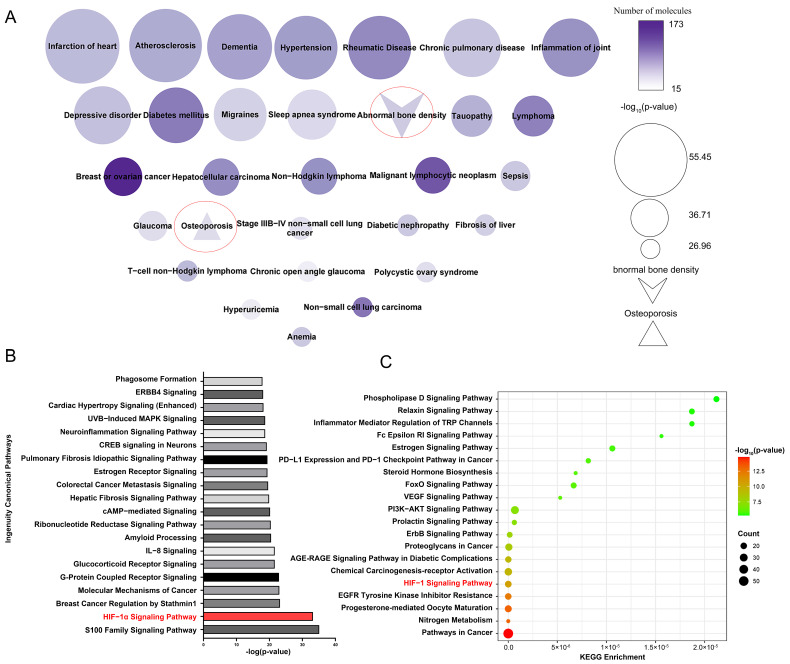
Enriched pathways and diseases related to the potential target proteins. (**A**) Diseases related to potential target proteins, enriched by IPA. Node size reflects *p*-value significance, color intensity indicates the number of proteins. (**B**) Top 20 pathways related to potential target proteins, enriched according to IPA. (**C**) Top 20 pathways related to potential target protein, enriched by the KEGG pathway analysis tool.

SwissTargetPrediction identified 258 potential target proteins for 33 active compounds and 65 potential active compounds in *Epimedium*. The identified targets include 84 proteases, 61 kinases, 25 G protein-coupled receptors, 23 peptidases, 18 transporters, 14 ligand-dependent nuclear receptors, 10 phosphatases, 3 ion channels, 3 transcription regulators, 2 cytokines, 2 transmembrane receptors, and 13 proteins from other categories (Supplementary Table S4). Figure 2A displays a node map that visualizes the diseases regulated by the potential target proteins. The top 30 diseases included abnormal bone density and osteoporosis, demonstrating the reliability of our target identification method and highlighting the targets’ association with osteoporosis.

Ingenuity Pathway Analysis (IPA) enrichment analysis identified 487 pathways linked to the potential target proteins, which were ranked by significance according to their -log p values, with the top 20 pathways shown in Figure 2B. Additionally, the Kyoto Encyclopedia of Genes and Genomes (KEGG) pathway analysis tool was used to perform enrichment analysis on the same potential target proteins (Figure 2C). We analyzed the pathways identified by both the IPA and KEGG enrichment analyses. Among the top five enriched pathways, the HIF-1α signaling pathway was the only one that overlapped. Existing studies have reported the association between the HIF-1α signaling pathway and osteoporosis. Yan et al. [22] found that resveratrol can promote osteoblast formation by inhibiting the ROS/HIF-1α signaling pathway to treat hypoxia-induced osteoporosis. Meng et al. [23] found that HIF-1α in B cells can increase osteoclast formation while the deletion of HIF-1α in B cells can alleviate ovariectomized (OVX)-induced bone loss. Therefore, the HIF-1α signaling pathway is very likely the pathway regulated by *Epimedium* in exerting its anti-osteoporosis effects.

The HIF-1α signaling pathway comprises 38 potential target proteins, of which 13 have been identified as targets for osteoporosis (Supplementary Table S5). Among them, HIF-1α is the key transcription factor in the HIF-1α signaling pathway [24]. It is related to glycolytic metabolism and regulates the expression of IL-β, which is the inflammatory in the vertebrae bone tissues of patients with postmenopausal osteoporosis [25,26]. Therefore, we infer that HIF-1α is a promising target protein of the active compounds in *Epimedium* for the treatment of osteoporosis. And HIF-1α was selected for further experimental validation.

### 2.2. Identification of 2″-O-RhamnosylIcariside II as a Key Active Compound from Epimedium for Osteoporosis Treatment by Targeted Inhibition of HIF-1α: In Vitro Experimental Validation

#### 2.2.1. Identification of Active Compounds’ Non-Toxic Concentration from Epimedium

**Figure 3:**
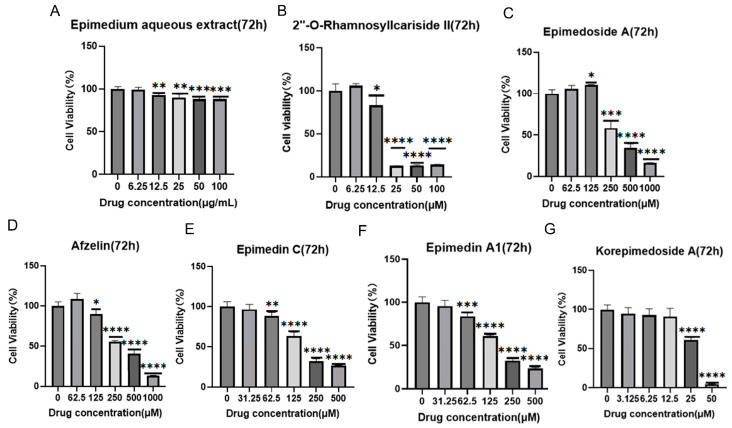
Effects of *Epimedium* aqueous extract and candidate monomers treatment for 72 h on the viability of MC3T3-E1 cells. Data shown as mean ± standard error of the mean (SEM) (n = 3). Statistical significance is indicated by * *p* < 0.05, ** *p* < 0.01, *** *p* < 0.001, and **** *p* < 0.0001 compared to the control group, which is at drug concentration of 0 μg/mL in panel (**A**) and 0 μM from panels (**B**–**G**).

It was reported that HIF-1α could alleviate osteoporosis by regulating osteoclast and osteoblast differentiation, with its inhibition shown to reduce osteoclast activity [27]. However, its role in osteoblast differentiation is still unclear, and some studies have suggested its inhibition promoted osteoblast differentiation and proliferation while others indicated its activation promoted osteoblast differentiation [28,29,30]. Therefore, we aimed to explore the potential osteoporosis treatment of 2″-O-RhamnosylIcariside II targeting HIF-1α in osteoblasts. Figure 3 displays the results of cell viability assays for *Epimedium* aqueous extract and six potential active compounds over 72 h, with each panel (A–G) corresponding to a different compound. The *Epimedium* aqueous extract and six other compounds were shown to inhibit the viability of MC3T3-E1 cells in a concentration-dependent manner. Notably, asterisks indicating statistical significance show substantial deviations from the control, implying that particular concentrations markedly influence the cell survival. The non-toxic concentration thresholds for these compounds were determined by examining the highest concentrations at which the cell viability remains unaffected after 72 h of treatment, as detailed in Table 2. Subsequently, we will investigate the effects of these compounds on cell differentiation at these concentrations, without impacting cell proliferation.

#### 2.2.2. Identification of Active Compounds from *Epimedium* That Influence Osteoblast Differentiation of MC3T3-E1 Cells under Hypoxic Conditions

HIF-1α is a transcription factor activated under hypoxic conditions and plays a central role in cellular adaptation to hypoxic environments, and which is rapidly degraded under normal oxygen levels and only stabilizes to influence gene expression under hypoxic conditions [31]. Several studies have reported that osteoclast and osteoblast are located in bone marrow cavities and epiphyseal, which are hypoxic areas, and their functions are regulated by the HIF-1α signaling pathway [31,32]. By simulating the pathological states, we can better understand the role of HIF-1α. Therefore, to investigate whether aqueous extract and six potentially active compounds from *Epimedium* enhance osteogenesis by inhibiting HIF-1α, it is necessary to establish a hypoxic environment.

**Figure 4:**
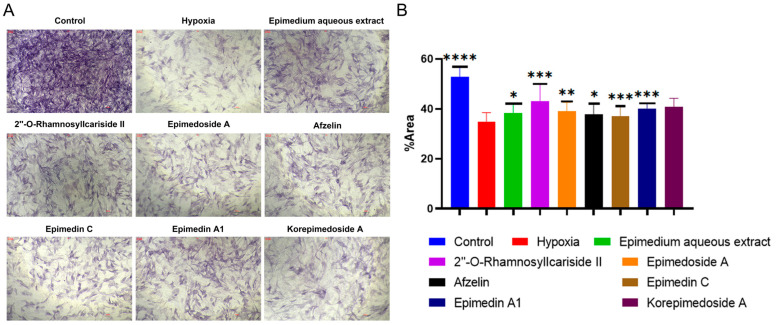
ALP staining results under hypoxic conditions. (**A**) ALP staining of MC3T3-E1 cells under hypoxic conditions, with the control group as the negative control. (**B**) The percentage of ALP positive areas in MC3T3-E1 cells under hypoxic conditions, data shown as mean ± SEM (n = 5). Scar bar is 200 μm. Statistical significance is indicated by * *p* < 0.05, ** *p* < 0.01, *** *p* < 0.001, and **** *p* < 0.0001 compared to the hypoxia group.

We conducted Alkaline Phosphatase (ALP) staining to assess the effect of potential active compounds in *Epimedium* on osteoblast differentiation with a MC3T3-E1 cell model. In the hypoxic environment, compared to the hypoxia group, the control group showed a significant increase in ALP activity, and treatments with *Epimedium* aqueous extract, 2″-O-RhamnosylIcariside II, Epimedoside A, Epimedin A1, and Korepimedoside A all significantly increased the ALP activity. Of these, the 2″-O-RhamnosylIcariside II treatment showed the most significant increase (Figure 4A,B, *p* < 0.05). These results showed that osteoblast differentiation was inhibited in hypoxia while aqueous *Epimedium* extract and the potential active compounds in *Epimedium*, except Korepimedoside A, could protect osteoblast differentiation in hypoxia, especially 2″-O-RhamnosylIcariside II.

#### 2.2.3. Identification of Active Compounds from *Epimedium* That Inhibit HIF-1α Gene Expression

**Figure 5:**
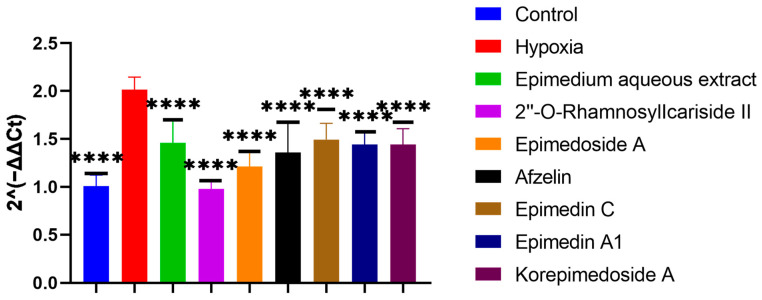
Effects of aqueous extract and potential active compounds on the expression of the HIF-1α gene during osteogenic differentiation of MC3T3-E1 Cells in hypoxia with the hypoxia group as the negative control. The internal reference was β-actin. Data shown as mean ± SEM (n = 3). Statistical significance is indicated by **** *p* < 0.0001 compared to the hypoxia group.

To screen which potential active compounds could increase the ALP activity of MC3T3-E1 cells through regulating HIF-1α gene expression in hypoxia, we conducted Reverse Transcription Real-time Quantitative Polymerase Chain Reaction (RT-qPCR) to study the expression of the HIF-1α gene during the osteogenic differentiation of MC3T3-E1 cells under hypoxic culture conditions. In hypoxia, the HIF-1α gene expression significantly increased in the hypoxia group compared to the control group. Further significant decreases were observed with the treatment group when compared to the hypoxia group. Notably, 2″-O-RhamnosylIcariside II showed the best inhibitory effect on HIF-1α gene expression and the best promoting effect on the ALP activity (Figure 4 and Figure 5, *p* < 0.05). Therefore, we selected 2″-O-RhamnosylIcariside II as the active compound with the most potential for further experimental validation based on the ALP staining and RT-qPCR results.

#### 2.2.4. Confirmation of 2″-O-RhamnosylIcariside II Existing in *Epimedium* Aqueous Extract Based on Mass Spectrometry Analysis

To confirm the presence of 2″-O-RhamnosylIcariside II in the *Epimedium* aqueous extract, we compared the mass spectrometry (MS) profiles—specifically retention time and m/z—of the extract against those of a 2″-O-RhamnosylIcariside II standard in positive ion mode. The analysis revealed retention times for the extract and the standard at 6.38 min and 6.48 min, with m/z values of 661.2479 for the extract and 661.24645 for the standard, respectively (Supplementary Figure S1A,B). These results definitively confirmed the presence of 2″-O-RhamnosylIcariside II in the *Epimedium* aqueous extract.

#### 2.2.5. Validation of HIF-1α as a Target Protein for 2″-O-Rhamnosylicariside II Using Bio-Layer Interferometry (BLI)

**Figure 6:**
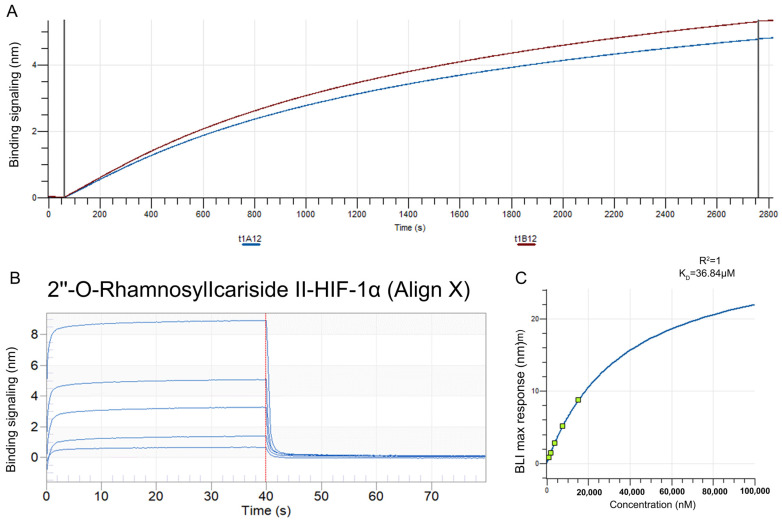
The data from BLI assays illustrating the interaction between 2″-O-RhamnosylIcariside II and HIF-1α protein. (**A**) The BLI signal intensity (nm) over time (s) when biotinylated HIF-1α was immobilized on an SA sensor. (**B**) Multiple cycles of binding and dissociation between 2″-O-RhamnosylIcariside II and HIF-1α. Each peak represents a binding event, where the vertical red dashed lines indicate the start and end points of the association and dissociation phases for each cycle. (**C**) The binding affinity curve, where the BLI response (nm) is plotted against the concentration of 2″-O-RhamnosylIcariside II (nM). The K_D_ was calculated by curve fitting the maximum BLI responses at different concentrations.

The BLI assay was conducted to explore the binding affinity between 2″-O-Rhamnosylicariside II and HIF-1α. A streptavidin (SA) sensor was used to immobilize biotinylated HIF-1α protein (10 μg/mL). The results showed that the immobilization signals from two repeated tests exceeded 4 nm, demonstrating successful biotinylation (Figure 6A). This helps to increase protein loading, thereby improving the success rate and accuracy of the BLI assay. Super streptavidin (SSA) sensors were then used to immobilize the HIF-1α protein for testing its binding affinity with 2″-O-RhamnosylIcariside II across a concentration range of 0.625–10 mg/mL. The results showed that 2″-O-RhamnosylIcariside II could bind with the HIF-1α protein, with the binding signal strength being positively correlated with the concentration (Figure 6B). A dynamic fitting curve indicated an affinity (K_D_) of 36.84 μM, supporting the hypothesis that HIF-1α is the target protein of 2″-O-rhamnosylirariside II (Figure 6C).

#### 2.2.6. 2″-O-RhamnosylIcariside II Can Inhibit the HIF-1α and Increase COL1A1 Protein Expression in MC3T3-E1 Cells Differentiation

COL1A1, which is known as collagen type I, serves as a crucial early biomarker for osteoblast differentiation and is the primary product of osteoblasts during bone formation [33,34]. HIF-1α is recognized as a pivotal regulator in hypoxic conditions and has been reported to inhibit the differentiation of osteoblasts [35]. To confirm that 2″-O-RhamnosylIcariside II enhances osteogenesis and treats osteoporosis by modulating the HIF-1α signaling pathway, Western blot and immunofluorescence assays were performed to assess the expression levels of COL1A1and HIF-1α before and after compound treatment.

**Figure 7:**
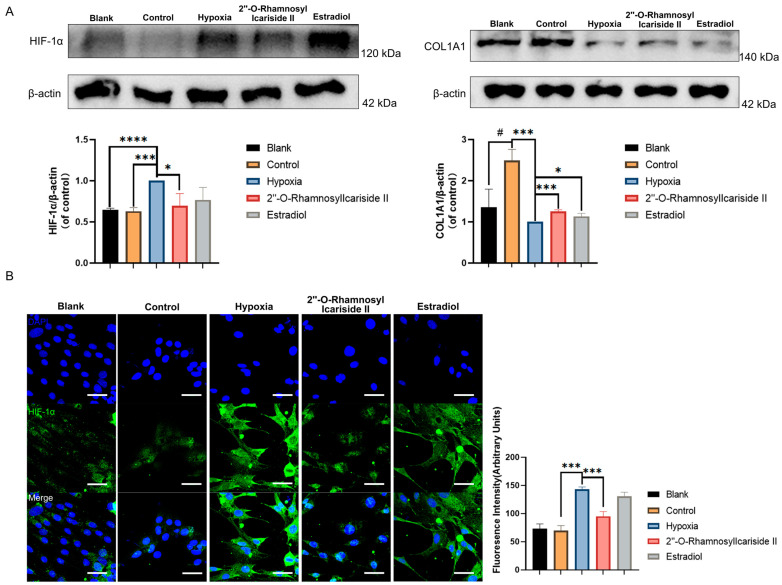
Relative protein expression levels of HIF-1α and COL1A1 during MC3T3-E1 cell osteogenic differentiation under hypoxic conditions. (**A**) Western blot results. (**B**) Immunofluorescence results. Blue fluorescence represents cell nuclei, while green fluorescence represents HIF-1α. The blank group was cultured in α-MEM medium. The blank group was treated by normal medium; the control group was cultured in induction medium; the hypoxia group was treated by osteogenic medium with 100 μM CoCl_2_, set as the negative group in hypoxic condition; the 2″-O-RhamnosylIcariside II group was treated by osteogenic medium with 100 μM CoCl_2_ and 6.25 μM 2″-O-RhamnosylIcariside II; and the Estradiol group was treated by osteogenic medium with 100 μM CoCl_2_ and 10 μM Estradiol. Scar bar is 50 μm. Data are presented as mean ± SEM (n = 3). ^#^ *p* < 0.05 indicates statistical significance compared to blank group, * *p* < 0.05, *** *p* < 0.001, **** *p* < 0.0001 indicate statistical significance compared to hypoxia group.

The Western blot results showed a significant increase in COL1A1 protein expression in the control group compared to the blank group, indicating effective osteogenic induction of MC3T3-E1 cells (Figure 7A, *p* < 0.05). After CoCl_2_-induced hypoxic cultivation of the hypoxia group, the COL1A1 protein expression was significantly decreased compared to the control group (Figure 7A, *p* < 0.05). Treatment with 2″-O-RhamnosylIcariside II or estrogen in hypoxia could significantly increase the COL1A1 protein expression compared to the hypoxia group, indicating the protective effect on osteoblast differentiation in hypoxia (Figure 7A, *p* < 0.05).

The western blot results also showed that HIF-1α protein levels in the hypoxia group were upgraded compared to the control group, while there was no significance in HIF-1α protein expression between the blank group and control group (Figure 7A, *p* < 0.05). And treatment with 2″-O-RhamnosylIcariside II significantly reduced HIF-1α protein expression, while estrogen treatment had no significant effect (Figure 7A, *p* < 0.05). The immunofluorescence assays exhibited similar results to the Western blot, showing a significant increase in the fluorescence intensity of the HIF-1α protein in the hypoxia group compared to the control group and no significance between the blank group and the control group (Figure 7B, *p* < 0.05). Subsequently, following treatment with 2″-O-RhamnosylIcariside II, there was also a significant decrease in the fluorescence intensity of the HIF-1α protein while estrogen treatment had no significant effect on regulating the HIF-1α protein in hypoxia (Figure 7B, *p* < 0.05). Therefore, these results indicate that, under hypoxic conditions, 2″-O-RhamnosylIcariside II can promote the osteogenic differentiation of MC3T3-E1 cells by promoting COL1A1 protein expression and inhibiting HIF-1α protein expression.

### 2.3. Validating 2″-O-RhamnosylIcariside II as an HIF-1α Inhibitor for Osteoporosis Treatment: In Vivo Experiments

#### 2.3.1. Assessment of In Vivo Safety of 2″-O-RhamnosylIcariside II

**Figure 8:**
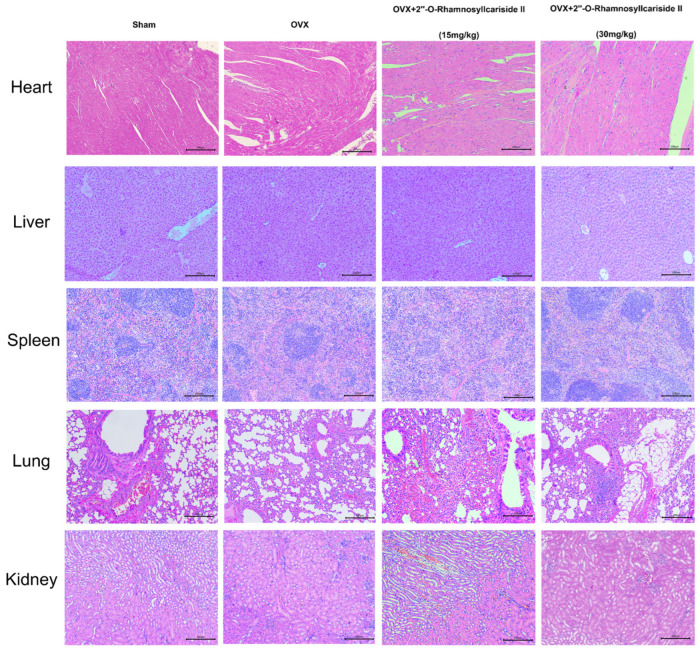
Histological analysis of mouse tissue samples for evaluating the in vivo safety of 2″-O-RhamnosylIcariside II. H&E staining across five different tissue types from Sham, OVX, and OVX mice treated with 2″-O-RhamnosylIcariside II at doses of 15 mg/kg and 30 mg/kg. Scar bar is 200 μm.

Figure 8 depicts the results of a histological analysis conducted on mouse tissue samples to evaluate the in vivo safety profile of 2″-O-RhamnosylIcariside II. Samples from the heart, liver, spleen, lung, and kidney across four groups—Sham, OVX, and OVX treated with 15 mg/kg and 30 mg/kg doses of 2″-O-RhamnosylIcariside II—have been stained using Hematoxylin and Eosin (H&E). The tissue sections from the Sham group serve as a normal control, showing the expected cellular architecture and histological features. Treatment with 2″-O-RhamnosylIcariside II at both dosages appears not to induce any additional histopathological changes, suggesting no overt toxicity in these vital organs. The preservation of tissue integrity in the treated groups is indicative of the compound’s safety at the administered doses for in vivo use in mice.

#### 2.3.2. 2″-O-RhamnosylIcariside II Can Alleviate Bone Loss of OVX Model

**Figure 9:**
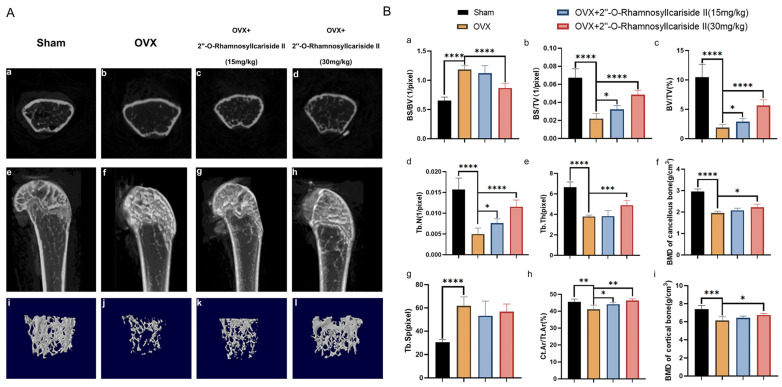
Therapeutic effects of 2″-O-RhamnosylIcariside II on bone microstructure in OVX mice. (**A**) Micro-CT assessments of femoral tissue morphology and bone histomorphometry in the OVX mouse model, showing both cross-sectional and 3D reconstructed images to illustrate the effects of 2″-O-RhamnosylIcariside II treatment on bone architecture. (a–d) Micro-CT images about cross section of femur. (e–h) Micro-CT images about longitudinal section of femur. (i–l) 3D images of microstructure of the distal femur trabecular bone. (**B**) Evaluation of the impact of 2″-O-RhamnosylIcariside II on trabecular bone histomorphometric and cortical bone histomorphometric parameters in the OVX model. (a) Statistic analysis of BS/BV (1/pixel). (b) Statistic analysis of BS/TV (1/pixel). (c) Statistic analysis of BV/TV (%). (d) Statistic analysis of Tb.N (1/pixel). (e) Statistic analysis of Tb.Th (pixel). (f) Statistic analysis of BMD of cancellous bone (g/cm^3^). (g) Statistic analysis of Tb.Sp (pixel). (h) Statistic analysis of Ct.Ar/Tt.Ar (%). (i) Statistic analysis of BMD of cortical bone (g/cm^3^). Data are presented as the mean ± SEM (n = 6); * *p* < 0.05, ** *p* < 0.01, *** *p* < 0.001, **** *p* < 0.0001 indicate statistical significance compared to the OVX group.

Micro-CT analysis was employed to evaluate the treatment effect of 2″-O-RhamnosylIcariside II on the cancellous bone and cortical bone of OVX mice by scanning the area 2 mm above the femoral epiphyseal and on the shaft from 1/3 to 1/2 mm below the femoral epiphyseal, respectively. The comparative Micro-CT imaging results depicted in Figure 9A(a,b,e,f,i,j) revealed a bone loss in the OVX group when contrasted with the Sham group, suggesting successful induction of the osteoporotic model. Subsequent administration of 2″-O-RhamnosylIcariside II can improve the bone microstructure deterioration (Figure 9A(b,d,f,h,j,l)).

Cancellous bone histomorphometric analyses, as shown in Figure 9B(a,g), revealed a significantly elevated bone surface area to tissue volume ratio (BS/BV) and trabecular separation (Tb.Sp) in the OVX group compared to the Sham group, indicating that the osteoporotic effects of ovariectomy led to a diminished bone density and a more disconnected trabecular network, hallmarks of deteriorated bone strength and structural integrity typically seen in osteoporosis. And a dose of 30 mg/kg of 2″-O-RhamnosylIcariside II significantly decreased the BS/BV, indicative of its osteoprotective effect (Figure 9B(a), *p* < 0.05). However, 2″-O-rhamnosylicariside II did not significantly affect the Tb.Sp, indicating that, while the compound has a beneficial impact on certain aspects of bone microarchitecture, it may not influence the overall spacing between trabeculae within the bone matrix (Figure 9B(g)).

Parameters of cortical bone and other parameters of cancellous bone, including the bone surface area to total volume ratio (BS/TV), the proportion of bone volume (BV/TV), the number of trabeculae (Tb.N), the trabecular thickness (Tb.Th), the bone mineral density (BMD) of cancellous bone, the ratio of the cortical bone area to the total area (Ct.Ar/Tt.Ar), and the BMD of cortical bone were significantly reduced in the OVX group compared to the Sham group (Figure 9B(b–f,h,i), *p* < 0.05). This reduction indicated a comprehensive decline in bone quality and robustness associated with osteoporotic progression due to estrogen deficiency post-ovariectomy. 2″-O-RhamnosylIcariside II was able to dose-dependently increase the BS/TV, BV/TV, Tb.N, and Ct.Ar/Tt.Ar, proving its therapeutic effect on osteoporosis (Figure 9B(b,d,h), *p* < 0.05). In addition, a 30 mg/kg dose of 2″-O-RhamnosylIcariside II significantly increased the Tb.Th, BMD of cancellous bone, and BMD of cortical bone, further indicative of its osteoprotective effect (Figure 9B(e,f,i), *p* < 0.05). In summary, 2″-O-RhamnosylIcariside II effectively improves both cancellous bone and cortical bone microstructures, thereby mitigating bone loss in the OVX mouse model.

#### 2.3.3. 2″-O-RhamnosylIcariside II Alleviates Weight Gain in OVX Mice

**Figure 10:**
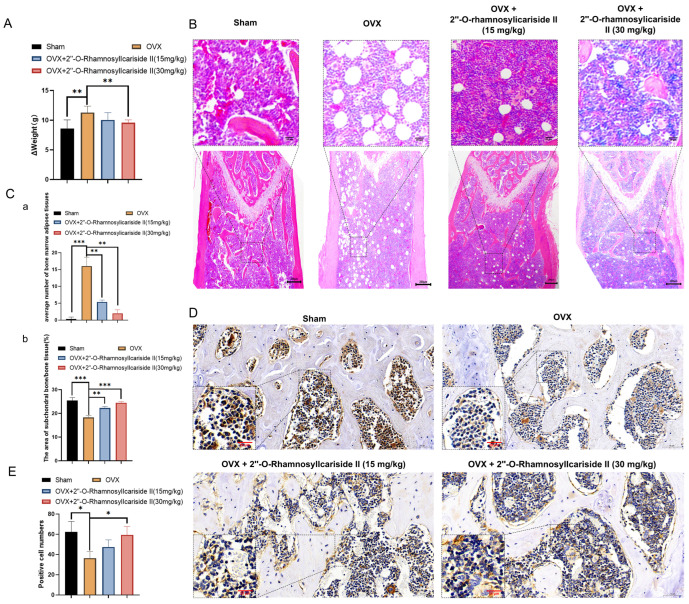
2″-O-RhamnosylIcariside II treated osteoporosis by inhibiting weight gain and promoting bone formation. (**A**) The effect of 2″-O-RhamnosylIcariside II on weight gain in the OVX mouse model. The data are presented as mean ± SEM (n = 6). ** *p* < 0.01 indicates statistical significance compared to the OVX group. (**B**) Analysis of the effects of 2″-O-RhamnosylIcariside II on femoral adipose tissues using H&E staining. Scar bar is 20 μm in partial enlarged images and 200 μm in original images, respectively. (**C**) Statistical analysis of the average number of bone marrow adipose tissues per unit area and the percentage of subchondral bone/bone tissue area in each group. (a) Statistic analysis of the area of subchondral bone/bone tissue (%). (b) Statistic analysis of positive cell numbers. Data are presented as the mean ± SEM (n = 6). ** *p* < 0.01, *** *p* < 0.001 indicate statistical significance compared to the OVX group. (**D**) Immunohistochemical analysis of 2″-O-RhamnosylIcariside II treatment on Osteocalcin (OCN) protein expression in the femur of OVX mice. Scar bar is 20 μm in partial enlarged images and 50 μm in original images, respectively. (**E**) Data are presented as the mean ± SEM (n = 3); * *p* < 0.05 indicate statistical significance compared to the OVX group.

Ovariectomy in mice reduces energy expenditure while their energy intake remains unchanged, leading to an energy surplus. This surplus is likely stored as increased adipose tissue mass, contributing to accelerated body weight gain [36]. As shown in Figure 10A, the weight gain, calculated by subtracting the pre-surgery weight from the weight at the end of the experiment, was significantly higher in the OVX group than in the Sham group. This observation is consistent with prior studies, confirming the successful establishment of the osteoporosis model. To evaluate the effect of 2″-O-RhamnosylIcariside II on body weight, we compared the body weight gain of each group. Notably, the weight gain was significantly reduced in the OVX group treated with 2″-O-RhamnosylIcariside II (30 mg/kg) compared to the untreated OVX group (Figure 10A, *p* < 0.05). These results indicated that 2″-O-RhamnosylIcariside II can alleviate OVX-induced weight gain in mice at a dosage of 30 mg/kg.

#### 2.3.4. 2″-O-RhamnosylIcariside II Improved Bone Microstructure and Reduced Bone Marrow Adipose Tissues in OVX Mice

Osteoporosis is often accompanied by the abnormal accumulation of bone marrow adipose tissues [37,38]. The H&E staining results revealed a significant increase in bone marrow adipose tissues in the OVX group compared to the Sham group (Figure 10B,C(a), *p* < 0.05). Additionally, 2″-O-RhamnosylIcariside II significantly reduced bone marrow adipose tissue in a dose-dependent manner compared to the OVX group (Figure 10C(a), *p* < 0.05). The H&E staining results also demonstrated that 2″-O-RhamnosylIcariside II significantly increased the subchondral bone trabeculae in a dose-dependent manner compared to the OVX group (Figure 10C(b), *p* < 0.05). These findings were consistent with previous Micro-CT analysis and weight assessment, suggesting that 2″-O-RhamnosylIcariside II effectively reduced bone marrow adipose tissues in the femur and improved the subchondral bone trabeculae in a dose-dependent manner, thereby treating osteoporosis.

#### 2.3.5. 2″-O-RhamnosylIcariside II Can Promote Bone Formation In Vivo

To explore the effect of 2″-O-RhamnosylIcariside II on bone formation in vivo, we utilized immunohistochemical staining to research the OCN protein expression, which is commonly known as the biomarker of bone formation [39]. The results showed that, compared to the Sham group, the OCN protein expression of the OVX group was significantly decreased (Figure 10D,E). Subsequently, treatment with 2″-O-RhamnosylIcariside II at 30 mg/kg could increase OCN expression, indicating the a protective effect on bone formation (Figure 10D,E).

#### 2.3.6. 2″-O-RhamnosylIcariside II Can Inhibit HIF-1α Protein Expression in Femur

**Figure 11:**
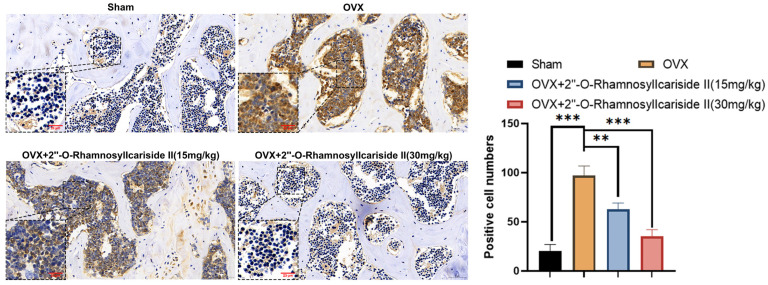
Immunohistochemical analysis of the effect of 2″-O-RhamnosylIcariside II treatment on HIF-1α protein expression in the femur of OVX mice. ** *p* < 0.01, *** *p* < 0.001 indicate a statistically significant difference compared to the Sham group. Scar bar is 20 μm in partial enlarged images and 50 μm in original images, respectively.

Immunohistochemical analysis was performed on femoral tissues to test their HIF-1α protein expression. The results indicated a significant increase in the number of positive cells for HIF-1α in the femoral bone of the OVX group compared to the Sham group, suggesting an upregulation of HIF-1α protein expression (Figure 11, *p* < 0.05). Additionally, 2″-O-RhamnosylIcariside II significantly decreased the number of positive cells in the femoral bone in a dose-dependent manner compared to the OVX group (Figure 11, *p* < 0.05). Therefore, 2″-O-RhamnosylIcariside II can treat osteoporosis in vivo by inhibiting the expression of the HIF-1α protein in the femoral bone.

#### 2.3.7. 2″-O-RhamnosylIcariside II Can Decrease the Number of Osteoclasts in the Femur of OVX Model

**Figure 12:**
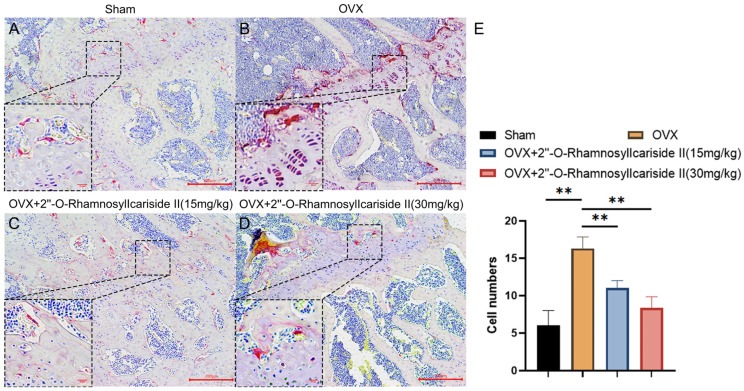
TRAP staining analysis of the effect of 2″-O-RhamnosylIcariside II treatment on osteoclasts in vivo. (**A**) Sham group. (**B**) OVX group. (**C**) OVX + 2″-O-RhamnosylIcariside II (15 mg/kg) group. (**D**) OVX + 2″-O-RhamnosylIcariside II (30 mg/kg) group. (**E**) The number of osteoclasts per unit area in each group. Scar bar is 20 μm in partial enlarged images and 200 μm in original images, respectively. The data are presented as the mean ± SEM (n = 3), ** *p* < 0.01 indicates a statistically significant difference compared to the OVX group.

We conducted tartrate-resistant acid phosphatase (TRAP) staining experiments on mouse femurs to explore the effect of 2″-O-RhamnosylIcariside II on osteoclast numbers in vivo. The results showed that estrogen deficiency significantly increased the number of osteoclasts in the OVX group (Figure 12A,B,E, *p* < 0.05). Following treatment with 2″-O-RhamnosylIcariside II at 15 mg/kg and 30 mg/kg significantly reduced the number of osteoclasts compared to the OVX group. (Figure 12B,E, *p* < 0.05). Therefore, 2″-O-RhamnosylIcariside II can treat osteoporosis by inhibiting osteoclast numbers in vivo.

## 3. Discussion

*Epimedium*, a traditional Chinese herb, has gained attention for its flavonoids that inhibit the activity of osteoclasts, thereby slowing osteoporosis progression [40]. Additionally, extracts from *Epimedium* enhance bone metabolism by promoting osteoblast proliferation and differentiation, increasing bone density, and inhibiting osteoblast apoptosis [41]. However, only 36 compounds have been explicitly reported to have anti-osteoporosis effects (Supplementary Table S2). Therefore, the specific anti-osteoporosis compounds in *Epimedium* and their mechanisms of action are still unclear.

In this study, we found that *Epimedium* could exert anti-osteoporosis effects by 33 active compounds and 65 potential active compounds targeting 258 potential proteins, thereby modulating 488 pathways. Subsequent network analysis and analysis of the literature selected six potential active compounds as the representative bioactive compounds of *Epimedium*, and HIF-1α as their potential target protein in treating osteoporosis. In vitro experiments showed that 2″-O-RhamnosylIcariside II was most effective at promoting ALP activity and suppressing HIF-1α gene expression in the MC3T3-E1 cell model under hypoxia conditions. Further in vitro experiments demonstrated that 2″-O-RhamnosylIcariside II could bind with HIF-1α and promote the differentiation of MC3T3-E1 cells into osteoblasts by upgrading COL1A1 protein expression and reducing HIF-1α protein expression. In vivo experiments confirmed that 2″-O-RhamnosylIcariside II could alleviate bone loss, protect bone formation, and improve the histomorphometry parameters of both cancellous bone and cortical bone. It achieves these effects by reducing marrow adipose tissue in the femur, protecting osteoblast embedded in the compact bone, and inhibiting HIF-1α protein expression, thereby exerting anti-osteoporosis effects.

HIF-1, a key transcriptional regulator in the cellular response to hypoxia, consists of HIF-1α and HIF-1β subunits and regulates the expression of genes involved in hypoxic responses. The hypoxia pathway not only enables organisms to adapt to specific environments, like short-term hypoxia on plateaus under normal physiological conditions, but it also plays a crucial role in the onset and progression of various diseases, including osteoporosis [42]. Bone, a unique organ of the body, exists in a relatively low-oxygen environment where the expression of HIF-related molecules sustains the essential conditions for bone development [42]. In 1999, it was first reported that the BMD of rats under hypoxic conditions was lower than that under normoxic conditions, suggesting a potential link between hypoxia and bone loss [43]. In addition, Yang et al. found that prenatal hypoxia increased OVX-induced osteoporosis in the elder offspring by inhibiting the IGF1 signaling pathway and extracellular matrix synthesis [44]. It was also reported that Isoquercetin could improve the histological characteristics of OVX-induced osteoporosis by inhibiting HIF-1α and increasing β-catenin expression, which is biomarker of osteoblast differentiation [45]. Therefore, the hypoxia pathway is closely linked to osteoporosis, necessitating further research into the mechanisms by which hypoxia can treat this condition, with the goal of discovering new therapeutic approaches.

HIF-1α has been reported to influence osteoclast differentiation and the osteogenic differentiation of BMSCs [46,47]. Many studies have shown that inhibition of HIF-1α can reduce osteoclast activity, thereby exerting an anti-osteoporosis effect. For example, Morita et al. [48] reported that selective estrogen receptor modulators like tamoxifen, raloxifene, and bazedoxifene can suppress osteoclast activity by inhibiting HIF-1α protein expression. However, the role of HIF-1α in either activating or inhibiting osteogenic activity during the treatment of osteoporosis varies, as reported by different studies with inconsistent results. For example, Chen et al. [49] found that HIF-1α inhibits the Wnt signaling pathway, thereby suppressing osteoblast proliferation and activity. This indicates that the inhibition of HIF-1α may promote osteoblast proliferation and activity, contributing to an anti-osteoporotic effect. Conversely, Li et al. [50] reported that raloxifene significantly increased the ALP staining intensity in osteoblasts by upregulating the protein expression of HIF-1α and β-catenin in OVX rat bone tissue. This indicates that the anti-osteoporotic effect of raloxifene is associated with the activation of HIF-1α and enhanced osteoblast proliferation, which challenges the findings of Chen et al. Given these conflicting outcomes, the role of the HIF-1α signaling pathway in osteogenic activity during osteoporosis treatment requires further investigation. Therefore, in this study, we focused on investigating the osteoporosis treatment effects of 2″-O-RhamnosylIcariside II by targeting HIF-1α in osteoblasts.

Although we have not systematically studied the effects of 2″-O-RhamnosylIcariside II in treating osteoporosis by targeting HIF-1α in osteoclasts, we preliminarily found that 2″-O-RhamnosylIcariside II significantly inhibited the number of osteoclasts both in vitro and in vivo in a dose-dependent manner by TRAP staining analysis (Supplementary Figure S2 and Figure 12). Further experiments are needed to confirm the potential therapeutic effects of 2″-O-RhamnosylIcariside II on osteoporosis by regulating HIF-1α.

This study is the first to identify that 2″-O-RhamnosylIcariside II can treat osteoporosis by specifically targeting and inhibiting HIF1-α in osteoblasts. These findings lay a scientific foundation for using *Epimedium* in the clinical treatment of osteoporosis and introduce a new candidate drug for this condition. Furthermore, this research contributes new evidence supporting HIF1-α as a viable therapeutic target for osteoporosis.

## 4. Materials and Methods

### 4.1. Materials

The *Epimedium* herbal medicine was purchased from Leiyunshang Pharmaceutical Co., Ltd., Shanghai, China. MC3T3-E1 cells were both acquired from the Cell Bank of the Chinese Academy of Sciences. Phosphate-buffered saline (PBS) was obtained from Biosharp (Cat#BL601A, Beijing, China). Penicillin-Streptomycin (10,000 U/mL, Cat#15140122), Trypsin-EDTA (0.25%) with phenol red (Cat#25200072) were purchased from GIBCO (New York, NY, USA). Fetal bovine serum from Australia was obtained from Avantor (Radnor, PA, USA, Cat#76294-180). α-MEM medium was purchased from Cytiva (Logan, UT, USA, Cat# SH30265.01). CoCl_2_ was obtained from Adamas (Basel, Switzerland, Cat#81782F). 2″-O-Rhamnosylicariside II (Cat#ST18550105), Epimedoside A (Cat#ST18360110), Afzelin (Cat#ST11030305), Epimedin C (Cat#ST00790120), Epimedin A1 (Cat#ST14860120), and Korepimedoside A (Cat#STG4990105) were all purchased from NatureStandard (Shanghai, China). The 4% paraformaldehyde universal tissue fixative was obtained from Biosharp (Beijing, China, Cat#BL539A). CCK-8 Kit was purchased from TEYE (Shanghai, China, Cat#TY0312). BCIP/NBT Alkaline Phosphatase Color Development Kit was purchased from Beyotime (Henan, China, Cat#C3206). TRIZOL was obtained from Ambion (Austin, TX, USA, Cat#155960-18). HiScript^®^ III RT SuperMix for qPCR was purchased from Vazyme (Shanghai, China, Cat#R323-01). The 2× SYBR Green qPCR Premix (Universal) was purchased from TEYE (Shanghai, China, Cat#TY0602). HIF-1α (Cat#20960-1-AP), COL1A1 (Cat# 67288-1-Ig), β-actin antibodies (Cat#81115-1-RR), HRP-conjugated affinipure goat anti-rabbit IgG(H+L) (Cat#SA00001-2), and fluorescein (FITC)-conjugated affinipure goat anti-rabbit IgG(H+L) (Cat#SA00003-2) were purchased from Proteintech (Wuhan, China). Primes of RT-qPCR were purchased from TsingkeBiotech (Beijing, China). For H&E staining, ethanol (Cat#100092683), xylene (Cat#10023418), normal butanol (Cat#100052190), and neutral gum (Cat#10004160) were purchased from Sinopharm Chemical Reagent Co., Ltd. (Shanghai, China), and environmentally friendly dewaxing transparent liquid (Cat#G1128-1L), paraformaldehyde fixative (Neutral, Cat#G1101), and H&E HD constant dye kit (Cat#G1076) were purchased from Servicebio (Wuhan, China). For immunohistochemistry staining, ethanol (Cat#100092683), xylene (Cat#10023418), normal butanol (Cat#100052190), and hydrochloric acid (Cat#10011028) were purchased from Sinopharm Chemical Reagent Co., Ltd. (Shanghai, China), 3% hydrogen peroxide disinfectant was purchased from Annjet High-Tech Disinfection Technology Co., Ltd. (Dezhou, China), and 20× citric acid antigen repair solution (pH 6.0, Cat# G1202), PBS buffer solution (Cat#G0002), paraformaldehyde fixative (Neutral, Cat#G1101), bovine serum albumin (Cat#GC305010), normal rabbit serum (concentrated, Cat#G1209), hematoxylin (Cat#G1004), differentiation fluid (Cat#G1039), ammonia (Cat#G1040), mounting medium (Cat#G1404), and histochemical reagent kit DAB chromogenic agent (Cat#G1212), OCN antibody (Cat#GB11233), and HRP-conjugated affinipure goat anti-rabbit IgG (Cat#GB23303) were all purchased and used from Servicebio (Wuhan, China). For TRAP staining, ethanol (Cat#100092683), xylene (Cat#10023418), and neutral gum (Cat#10004160) were purchased from Sinopharm Chemical Reagent Co., Ltd. (Shanghai, China), and environmentally friendly dewaxing transparent liquid (Cat#G1128), TRAP dye solution kit (Cat#G1050), hematoxylin (Cat#G1004), differentiation fluid (Cat#G1039), and ammonia (Cat#G1040) were all purchased and used from Servicebio (Wuhan, China).

### 4.2. Construction of the Epimedium Chemical Database

The *Epimedium* chemical database was constructed by retrieving data from the Traditional Chinese Medicine Integrated Database (TCMID, http://www.megabionet.org/tcmid, accessed on 10 October 2022), the Traditional Chinese Medicine Database@Taiwan (TCM Database@Taiwan, http://tcm.cmu.edu.tw, accessed on 12 October 2022), and the Chemistry Database (http://www.chemcpd.csdb.cn/scdb, accessed on 17 October 2022). This database contains information such as names, CAS numbers, and classification information. Subsequently, an extensive process of search, verification, consolidation, correction, and supplementation were conducted using the relevant literature, books, and the PubChem database (https://pubchem.ncbi.nlm.nih.gov, accessed on 23 October 2022) to ensure the accuracy and completeness of the database.

### 4.3. Construction of a Database with Known Anti-Osteoporosis Compounds in Epimedium

The PubMed database (https://pubmed.ncbi.nlm.nih.gov, accessed on 24 October 2022) was queried with the terms: (((osteoporosis) OR (postmenopausal osteoporosis)) OR (bone)) AND (compound name in the *Epimedium* chemical database). Compounds with anti-osteoporosis activity were filtered out, and their mechanisms of action against osteoporosis were compiled and summarized.

### 4.4. Construction of a Potential Active Compound Interaction Network Based on Molecular Similarity Calculations

The Open Babel tool was employed for converting compound formats from SMILES to SDF. Following this, the Rdkit package (https://www.rdkit.org/, accessed on 7 October 2022) in Python was used to generate the extended-connectivity fingerprint 4 (ECFP4) for the compounds. ECFP4 is a type of molecular fingerprint used in cheminformatics and computational chemistry. It is based on the concept of circular fingerprints, where each atom in a molecule is assigned a unique identifier based on its local chemical environment within a defined radius. ECFP4 specifically considers up to four iterations of atom connectivity, capturing information about chemical substructures within a molecule. T_c_ is a metric used to gauge the similarity between two sets or vectors, denoted by A and B. It is computed using the formula:

$$T_{c}\left(A,B\right)=\frac{A\cdot B}{{\left\|A\right\|}^{2}+{\left\|B\right\|}^{2}-A\cdot B}$$

In the context of this formula, A and B represent the ECFP4 of compound A and B, respectively. T_c_ is a metric that varies between 0 and 1, where 0 indicates no similarity and 1 indicates identical sets.

Subsequently, the panda package was used to save the data. Compounds with T_c_ of 0.4 or higher were identified as candidates likely to exhibit similar biological activities. Finally, an interaction network of potential compounds was constructed using Cytoscape software, version 3.8.1. In this network, compounds are connected by edges if their corresponding T_c_ surpasses the threshold of 0.4. The related code is available on Github (https://github.com/donghuizhong/DTI, accessed on 20 October 2023).

### 4.5. Prediction of Potential Target Proteins and Pathway Enrichment Analysis

The SwissTargetPrediction website (http://www.swisstargetprediction.ch/, accessed on 8 November 2022) was used to predict the potential target proteins for the compounds. Proteins with an average score greater than 0.1 were selected as potential targets [51]. Moreover, given that over 85% of the compounds in *Epimedium* share structural similarities with at least two other compounds, a criterion was established such that only target proteins associated with more than two related compounds were considered, in order to refine the precision of the target prediction. Finally, pathway enrichment analysis for the potential target proteins was conducted using both IPA software (www.qiagenbioinformatics.com, accessed on 5 December 2023) and KEGG pathway analysis (https://david.ncifcrf.gov/, accessed on 20 December 2023). In addition, disease enrichment analysis associated with these target proteins was also performed using the IPA software. Finally, Cytoscape software (version 3.8.1) and a bioinformatic website (https://www.bioinformatics.com.cn/, accessed on 25 December 2023) were used to graphic construction.

### 4.6. Preparation of the Aqueous Epimedium Extract

Five hundred grams of *Epimedium* were weighted out and soaked in 2 L cold water for one hour. Subsequently, an additional 1 L cold water was added to the soaked herb, and the mixture was boiled over medium heat. The mixture was boiled and stirred for approximately 40 min until it reduced to about 100 mL. The extract was freeze-dried, and then ground into a fine powder. This powdered extract was then stored for subsequent experimental use.

### 4.7. Cell Culture and Construction of Cellular Hypoxia Model

MC3T3-E1 cells were cultured in α-MEM supplemented with 10% fetal bovine serum and 1% penicillin/streptomycin inside a constant-temperature incubator maintained at 37 °C and 5% CO_2_. The culture medium for these cells was changed every 3–4 days. Upon reaching about 90% confluency, the MC3T3-E1 cells were passaged. To simulate cellular hypoxia, cells were treated with 100 μM CoCl_2_ solution for 24 or 72 h [52].

### 4.8. Cell Viability Experiments

MC3T3-E1 cells were seeded in a 96-well plate and given a 24-h period to adhere to the plate surface. Subsequently, we established blank, control, and treatment groups. The cells in treatment groups were treated with different concentrations of drugs. The blank group consists of wells containing only the culture medium without any cells or drugs, serving as a baseline to measure the background signal in the assays. The control group includes cells maintained under identical conditions to the treatment group but without the addition of drugs, serving to illustrate the cells’ behavior under normal conditions for comparison against the effects observed in the treatment group. Each group set contained 3 replicate wells. After culturing for 72 h, CCK-8 Kit was used to evaluate the cell viability. Absorbance at 450 nm was measured using a microplate reader. The formula used to calculate the relative cell viability for each group is:

$$\frac{\left(A_{Treatment\;group}\right)-(A_{Blank\;group})}{\left(A_{Control\;group}\right)-(A_{Blank\;group})}\times 100\%$$

### 4.9. ALP Staining

MC3T3-E1 cells were seeded in a 96-well plate and allowed 24 h to adhere to the plate surface. Subsequently, we set control, hypoxia, and treatment groups (Table 3). Hypoxic condition was simulated in hypoxia and treatment groups by adding 100 μM CoCl_2_. Normal medium indicates the basic culture medium without any additives. The osteogenic induction medium was formulated by supplementing normal medium with 0.01% dexamethasone, 0.2% ascorbic acid, and 1% β-glycerol phosphate, and the preparation was carried out under dark conditions. The medium was changed every 3 days across a 7-day culture period. For hypoxia group, on day four, 100 μM CoCl_2_ was added to hypoxia and treatment groups to establish hypoxic conditions. Post-cultivation, BCIP/NBT staining solution was added to the cells, which were then incubated in darkness at room temperature for from one to two hours. An inverted microscope was used to observe the staining effect.

### 4.10. RT-qPCR Experiment

The cell culture and grouping are the same as above ALP staining, except that 100 μM CoCl_2_ was added to both the hypoxia and treatment groups to simulate hypoxic condition on the sixth day. TRIZOL was used to extract total RNA, after which chloroform and isopropanol were added, and the precipitate was collected by centrifuging at 14,000× *g* for 15 min at 4 °C. RNA was qualified by NanoDrop^™^ One/One^C^ (Thermo Fisher Scientific, Waltham, MA, USA). The RNA samples were then prepared with preheated DEPC-treated water. cDNA was obtained by reverse transcription kit, and PCR was then performed to amplify the target sequences. Each cDNA sample was diluted with DEPC-treated water to 1/5 of original concentration and amplified using a mixture that included 7.5 μL of SYBR Green qPCR Premix, 1 μL of upstream primer, 1 μL of downstream primer, 3.5 μL of DEPC-treated water, and 2 μL of the cDNA template (Table 4). The qPCR cycling conditions involved an initial denaturation at 95 °C for 3 min, followed by 40 cycles of 10 s at 95 °C for denaturation and 10 s at 60 °C for annealing and extension. A melt curve analysis was performed with the following steps: 5 s at 65 °C, 1 min at 60 °C, and a final denaturation at 95 °C for 1 s to record the dissolution curve.

### 4.11. BLI Technology

The HIF-1α recombinant protein was dissolved in sterile double-distilled water to achieve a concentration of 1 mg/mL. The HIF-1α protein concentration was tested by BCA assays to obtain the actual concentration before and after protein dialysis. The desalination was conducted via dialysis bag with a buffer containing PBS with 0.02% Tween 20. The dialysis buffer was changed every 4–6 h over a total period of 12–24 h. This process was conducted at 4 °C to prevent protein denaturation. A biotinylation solution of NHS-PEG12-Biotin was prepared at 10 mM, and then the protein was biotinylated by mixing it with the biotinylation reagent in a 3:1 ratio. The biotinylation reaction proceeded at room temperature for 45 min. Any unbound biotin was removed using a PD MiniTrapTM G-25 Desalting Column, after which the protein concentration was measured again. The protein was diluted to 10 μg/mL for immobilization on SA sensors, which were prewetted for at least 10 min prior to use. Four SSA sensors were also prewetted with PBS, with two designated for immobilizing the biotinylated protein and the other two serving as reference sensors. The detection protocol comprised a 30 s baseline period, followed by 40 s phases for both association and dissociation, and, to conclude, a brief 9.99 s equilibrium phase. This sequence was repeated to test a range of small molecule concentrations sequentially. Octet Analysis Studio (Ver. 12.2) was used to analyze the association and dissociation curves to determine the binding kinetics, thus calculating the binding affinity between the compound and the protein.

### 4.12. Quality Control of 2″-O-RhamnosylIcariside II

Samples, including 10 mM 2″-O-RhamnosylIcariside II and 10 mg/mL *Epimedium* water extract (both dissolved in DMSO), were separated on a Vanquish Core HPLC system (Thermo Fisher Scientific, Waltham, MA, USA) equipped with an XSelect HSS T3 column (2.5 μm, 2.1 mm × 100 mm, Waters Corporation, Milford, MA, USA). Analysis was carried out with a Q Exactive Plus Orbitrap mass spectrometer (Thermo Fisher Scientific, Waltham, MA, USA). The column temperature was set at 40 °C. The mobile phase consisted of 0.1% formic acid in water (Solvent A) and 0.1% formic acid in acetonitrile (Solvent B). The elution gradient was as follows: 0–7 min, 1% to 95% B; 7–10 min, 95% B; and 10–11 min, 95% to 1% B. The flow rate was 0.3 mL/min, and the injection volume was 2 μL. The mass-to-charge (m/z) collection range spanned from 100 to 1500.

### 4.13. Western Blot Analysis

MC3T3-E1 cells were seeded in a 6-well plate and allowed 24 h to adhere to the plate surface. Subsequently, we set four groups: blank, control, hypoxia, and treatment (2″-O-rhamnosylirariside II and estradiol) in hypoxia (Table 5). The medium was changed every three days across a seven-day culture period. On day six, the treatment and hypoxia groups were exposed to 100 μM CoCl_2_ and incubated for 24 h. Following cell culture, protein extraction was performed using RIPA lysis buffer containing 10% PMSF protease inhibitor. The protein samples were then subjected to sonication, followed by BCA assay, and boiled with loading buffer for 10–15 min. Electrophoresis was performed at 80 V for 30 min for the concentration gel and at 120 V for 100 min for the separation gel. Post-electrophoresis, proteins were transferred to a membrane via wet transfer, which was then blocked with a protein-free rapid blocking solution for 2 h at room temperature. Overnight immunoblotting at 4 °C was conducted using primary antibodies against HIF-1α (rabbit anti-goat, 1:2000), COL1A1 (rabbit anti-goat, 1:1000), and β-actin (rabbit anti-goat, 1:5000) as the internal reference. The membrane was rinsed with TBST and incubated with anti-rabbit secondary antibodies (1:2000) at room temperature for 1 h. Following three 5-min washes with TBST, protein bands were visualized by a chemiluminescence imaging system.

### 4.14. Immunofluorescence Analysis

Cell grouping and drug treatment are consistent with those described in above Western blot experiments. Following the treatment, cells were washed gently with PBS twice, then fixed with 4% paraformaldehyde for 20 min. Subsequently, the cells were treated with Triton X-100 for 10 min to disrupt the cell membrane. Afterward, they were incubated with HIF-1α primary antibody (1:200) overnight at 4 °C, followed by incubation with a FITC-conjugated secondary antibody (anti-rabbit, 1:100) at room temperature in the dark for 1 h. Finally, the cells were stained with DAPI for 10 min in the dark. Confocal microscopy was employed to capture images for further analysis.

### 4.15. Animal Experiments

Twenty-four 4-week-old female C57BL/6 mice were randomly divided into four groups: Sham, OVX, OVX treated with 15 mg/kg of 2-O-RhamnosylIcariside II, and OVX treated with 30 mg/kg of 2-O-RhamnosylIcariside II. After one week of acclimatization, Sham-operated mice underwent a procedure to remove adipose tissue surrounding the ovaries while under anesthesia. Mice in the OVX and treatment groups received ovariectomies under anesthesia and were subsequently weighed. The 2″-O-RhamnosylIcariside II solution was prepared using physiological saline containing 15% polyethylene-hydrogenated castor oil and 6% DMSO, and subsequently sonicated. Drug administration began one week after surgery, with the OVX group receiving saline plus 15% polyethylene hydrogenated castor oil and the treatment groups receiving 2″-O-RhamnosylIcariside II solution at 15 mg/kg and 30 mg/kg, respectively. Treatments were administered every other day for two months. Twenty-four hours after the last treatment, the mice were weighed again, and then tissues including the heart, liver, spleen, lung, kidney, and limbs were fixed with 4% paraformaldehyde for subsequent histopathologic analysis. All the animal experiments received approval from the ethics committee of Shanghai University.

### 4.16. Histopathologic Analysis

The heart, liver, spleen, lung, kidney, limb, and femoral tissue samples were first embedded in paraffin, and then sectioned to the desired thickness. These paraffin-embedded sections were then subjected to H&E staining, which provides a detailed view of the tissue structure and the cellular components within. Methylene blue-acid fuchsin staining was carried out in femoral tissue samples, which is particularly useful for identifying osteoblasts and assessing bone formation. Additionally, TRAP staining was carried out in femoral tissue samples, which is particularly useful for identifying osteoclasts. Immunohistochemistry staining was also performed to detect specific proteins and antigens within the tissues. These processes were executed by the professional services of Servicebio (Servicebio Biotechnology Co., Ltd., Wuhan, China).

### 4.17. Micro-CT and Histomorphometric Analysis

The femoral tissues were fixed in 4% paraformaldehyde for 24 h and then secured onto the micro-CT scanner platform. A comprehensive 360-degree scan of the specimens was conducted. The scanning parameters were set as: 143 μA current, 70 V voltage, 0.2-degree rotation step, and 9 μm image resolution. For trabecular bone, we chose 100 slices 2 mm above the growth plate. For cortical bone, we chose 100 slices in the middle part of the femur. After scanning, the bone histomorphometric parameters were analyzed using CTAn software (Ver. 1.17.7.2), followed by a three-dimensional reconstruction of the scanned images with CTvol software (Ver. 2.3.2.0). The threshold of trabecular bone and cortical bone was set at 40 and 80, respectively. CTvox software (Ver. 3.3.0) was further used to process the reconstructed image.

### 4.18. Statistical Analysis

Microsoft Paint was used for image editing, and Image J software (Ver. 1.51) was employed for analyzing the grayscale intensities of Western Blot bands. GraphPad Prism 8.0.2 software was utilized for performing statistical analysis and visualizing the scientific data. The data are presented as mean values with SEM. For the data analysis, an unpaired *t*-test was used for comparing two sample groups. When comparing multiple sample groups, one-way analysis of variance (ANOVA) method was applied. If significant differences were identified, Tukey’s post-hoc test was conducted for pairwise group comparisons. A *p*-value below 0.05 was considered to be statistically significant.

### 4.19. Ethics Statement

All animal studies and procedures have been approved by Ethics Committee of Shanghai University and performed in accordance with the ethical standards. The ethical approval code was ECSHU2024-014.

## 5. Conclusions

In summary, network pharmacology analysis and in vitro experiments revealed that 2″-O-RhamnosylIcariside II, a key active compound of *Epimedium*, enhanced COL1A1 protein expression and ALP activity, thereby promoting osteogenic differentiation by targeting and inhibiting HIF-1α under hypoxia condition. In vivo experiments showed that 2″-O-RhamnosylIcariside II can exert an anti-osteoporosis effect by alleviating bone loss, promoting bone formation, and inhibiting HIF-1α protein expression. This study establishes a scientific foundation for the clinical use of *Epimedium* and introduces a new potential drug for treating osteoporosis. Furthermore, it offers fresh evidence supporting HIF-1α as a therapeutic target for osteoporosis.

## Figures and Tables

**Table 1:** Potential active compounds of *Epimedium*.

Candidate Compound	CAS (Chemical Abstracts Service Number)	Tc Similarity
2″-O-Rhamnosylicariside II	135293-13-9	0.65983897
Epimedoside A	39012-04-9	0.650410123
Afzelin	482-39-3	0.630783002
Epimedin C	110642-44-9	0.624386306
Epimedin A1	140147-77-9	0.596296546
Korepimedoside A	106441-31-0	0.502801148

**Table 2:** The optimal concentration of aqueous extract and potential active compounds for cell treatment.

Compound	Concentration for MC3T3-E1
*Epimedium* aqueous extract	6.25 μg/mL
2″-O-RhamnosylIcariside II	6.25 μM
Epimedoside A	62.5 μM
Afzelin	62.5 μM
Epimedin C	31.25 μM
Epimedin A1	31.25 μM
Korepimedoside A	12.5 μM

**Table 3:** The experimental conditions applied to MC3T3-E1 cells across different groups under hypoxic conditions in ALP staining and RT-qPCR assays. Checkmarks (✓) indicate the application of the respective condition to the cell groups.

	Control	Hypoxia	Treatment
Induction medium	✓	✓	✓
Treatment			✓
CoCl_2_ (100 μM)		✓	✓

**Table 4:** The primer sequences used for PCR amplification of genes.

Gene	Primer Sequence (5′→3′)
Mouse Hif1a	Upstream: GTGCACAGAGCCTCCTCG
	Downstream: AGCTGGAAGGTTTGTGGTGT
Mouse Atcb	Upstream: AGCCATGTACGTAGCCATCC
	Downstream: GACTCCATCACAATGCCAGT

**Table 5:** The experimental conditions applied to MC3T3-E1 cells across different groups under hypoxic conditions in Western blot experiments. Checkmarks (✓) indicate the application of the respective condition to the cell groups.

	Blank	Control	Hypoxia	Treatment
Normal medium	✓			
Induction medium		✓	✓	✓
Treatment				✓
CoCl_2_ (100 μM)			✓	✓

## Data Availability

All data were shared within the article.

## Supplementary Materials

The following supporting information can be downloaded at: https://www.mdpi.com/article/10.3390/ph17060706/s1, Table S1: Database of Chemical Constituents of *Epimedium*; Table S2: Known Anti-Osteoporosis Active Monomers from *Epimedium* and Their Mechanisms of Action; Table S3: Pairs of Potential Active Compounds with Tanimoto Similarity Greater Than 0.4 (The No. was consistent with the compounds represented in Supplementary Table S1); Table S4: Potential Target Proteins for *Epimedium* Against Osteoporosis; Table S5: Potential target proteins in HIF-1α Pathway; Figure S1: The presence of 2″-O-RhamnosylIcariside II in the aqueous *Epimedium* extract; Figure S2: TRAP staining analysis of the effect of 2″-O-rhamnosylicariside II on the ability of RAW264.7 cells to differentiate into osteoclasts.

## References

[1] Ensrud K.E., Crandall C.J. (2017). Osteoporosis. Ann. Intern. Med..

[2] Johnell O., Kanis J.A. (2006). An Estimate of the Worldwide Prevalence and Disability Associated with Osteoporotic Fractures. Osteoporos. Int..

[3] Tella S.H., Gallagher J.C. (2014). Prevention and Treatment of Postmenopausal Osteoporosis. J. Steroid Biochem. Mol. Biol..

[4] Johnston C.B., Dagar M. (2020). Osteoporosis in Older Adults. Med. Clin. N. Am..

[5] Shieh A., Ishii S., Greendale G.A., Cauley J.A., Lo J.C., Karlamangla A.S. (2016). Urinary N-Telopeptide and Rate of Bone Loss Over the Menopause Transition and Early Postmenopause. J. Bone Miner. Res..

[6] Gosset A., Pouillès J.-M., Trémollieres F. (2021). Menopausal Hormone Therapy for the Management of Osteoporosis. Best. Pract. Res. Clin. Endocrinol. Metab..

[7] Khosla S., Hofbauer L.C. (2017). Osteoporosis Treatment: Recent Developments and Ongoing Challenges. Lancet Diabetes Endocrinol..

[8] Quattrocchi E., Kourlas H. (2004). Teriparatide: A Review. Clin. Ther..

[9] Lamy O., Stoll D., Aubry-Rozier B., Rodriguez E.G. (2019). Stopping Denosumab. Curr. Osteoporos. Rep..

[10] Zhuo Y., Li M., Jiang Q., Ke H., Liang Q., Zeng L.-F., Fang J. (2022). Evolving Roles of Natural Terpenoids From Traditional Chinese Medicine in the Treatment of Osteoporosis. Front. Endocrinol..

[11] Zou Y., Wang S., Zhang H., Gu Y., Chen H., Huang Z., Yang F., Li W., Chen C., Men L. (2024). The Triangular Relationship between Traditional Chinese Medicines, Intestinal Flora, and Colorectal Cancer. Med. Res. Rev..

[12] Zhang X., Sun J., Wang J., Meng T., Yang J., Zhou Y. (2023). The Role of Ferroptosis in Diabetic Cardiovascular Diseases and the Intervention of Active Ingredients of Traditional Chinese Medicine. Front. Pharmacol..

[13] Liu Z.-Q., Sun X., Liu Z.-B., Zhang T., Zhang L.-L., Wu C.-J. (2022). Phytochemicals in Traditional Chinese Medicine Can Treat Gout by Regulating Intestinal Flora through Inactivating NLRP3 and Inhibiting XOD Activity. J. Pharm. Pharmacol..

[14] Guo C., He J., Song X., Tan L., Wang M., Jiang P., Li Y., Cao Z., Peng C. (2019). Pharmacological Properties and Derivatives of Shikonin-A Review in Recent Years. Pharmacol. Res..

[15] Guo B., Xiao P. (2003). Comment on main species of herba epimedii. Zhongguo Zhong Yao Za Zhi.

[16] Wang Z., Lou Y. (2004). Proliferation-Stimulating Effects of Icaritin and Desmethylicaritin in MCF-7 Cells. Eur. J. Pharmacol..

[17] Ma H., He X., Yang Y., Li M., Hao D., Jia Z. (2011). The Genus Epimedium: An Ethnopharmacological and Phytochemical Review. J. Ethnopharmacol..

[18] Indran I.R., Liang R.L.Z., Min T.E., Yong E.-L. (2016). Preclinical Studies and Clinical Evaluation of Compounds from the Genus Epimedium for Osteoporosis and Bone Health. Pharmacol. Ther..

[19] Xu H., Zhou S., Qu R., Yang Y., Gong X., Hong Y., Jin A., Huang X., Dai Q., Jiang L. (2020). Icariin Prevents Oestrogen Deficiency-Induced Alveolar Bone Loss through Promoting Osteogenesis via STAT3. Cell Prolif..

[20] Zhou L., Poon C.C.-W., Wong K.-Y., Cao S., Dong X., Zhang Y., Wong M.-S. (2021). Icariin Ameliorates Estrogen-Deficiency Induced Bone Loss by Enhancing IGF-I Signaling via Its Crosstalk with Non-Genomic ERα Signaling. Phytomedicine.

[21] Tsai K.-C., Huang Y.-C., Liaw C.-C., Tsai C.-I., Chiou C.-T., Lin C.-J., Wei W.-C., Lin S.J.-S., Tseng Y.-H., Yeh K.-M. (2021). A Traditional Chinese Medicine Formula NRICM101 to Target COVID-19 through Multiple Pathways: A Bedside-to-Bench Study. Biomed. Pharmacother..

[22] Yan C., Wang Z., Liu W., Pu L., Li R., Ai C., Xu H., Zhang B., Wang T., Zhang X. (2022). Resveratrol Ameliorates High Altitude Hypoxia-Induced Osteoporosis by Suppressing the ROS/HIF Signaling Pathway. Molecules.

[23] Meng X., Lin Z., Cao S., Janowska I., Sonomoto K., Andreev D., Katharina K., Wen J., Knaup K.X., Wiesener M.S. (2022). Estrogen-Mediated Downregulation of HIF-1α Signaling in B Lymphocytes Influences Postmenopausal Bone Loss. Bone Res..

[24] Warbrick I., Rabkin S.W. (2019). Hypoxia-Inducible Factor 1-Alpha (HIF-1α) as a Factor Mediating the Relationship between Obesity and Heart Failure with Preserved Ejection Fraction. Obes. Rev..

[25] Zeng C.-Y., Wang X.-F., Hua F.-Z. (2022). HIF-1α in Osteoarthritis: From Pathogenesis to Therapeutic Implications. Front. Pharmacol..

[26] Qiao S., Zhang X., Chen Z., Zhao Y., Tzeng C.-M. (2023). Alloferon-1 Ameliorates Estrogen Deficiency-Induced Osteoporosis through Dampening the NLRP3/Caspase-1/IL-1β/IL-18 Signaling Pathway. Int. Immunopharmacol..

[27] Knowles H.J., Cleton-Jansen A.-M., Korsching E., Athanasou N.A. (2010). Hypoxia-Inducible Factor Regulates Osteoclast-Mediated Bone Resorption: Role of Angiopoietin-like 4. FASEB J..

[28] Tian Y., Shao Q., Tang Y., Li X., Qi X., Jiang R., Liang Y., Kang F. (2022). HIF-1α Regulates Osteoclast Activation and Mediates Osteogenesis during Mandibular Bone Repair via CT-1. Oral. Dis..

[29] Frey J.L., Stonko D.P., Faugere M.-C., Riddle R.C. (2014). Hypoxia-Inducible Factor-1α Restricts the Anabolic Actions of Parathyroid Hormone. Bone Res..

[30] Wu Y., Wang M., Feng H., Peng Y., Sun J., Qu X., Li C. (2017). Lactate Induces Osteoblast Differentiation by Stabilization of HIF1α. Mol. Cell Endocrinol..

[31] Miyauchi Y., Sato Y., Kobayashi T., Yoshida S., Mori T., Kanagawa H., Katsuyama E., Fujie A., Hao W., Miyamoto K. (2013). HIF1α Is Required for Osteoclast Activation by Estrogen Deficiency in Postmenopausal Osteoporosis. Proc. Natl. Acad. Sci. USA.

[32] Wang Y., Wan C., Deng L., Liu X., Cao X., Gilbert S.R., Bouxsein M.L., Faugere M.-C., Guldberg R.E., Gerstenfeld L.C. (2007). The Hypoxia-Inducible Factor Alpha Pathway Couples Angiogenesis to Osteogenesis during Skeletal Development. J. Clin. Investig..

[33] Nakajima K., Kho D.H., Yanagawa T., Harazono Y., Gao X., Hogan V., Raz A. (2014). Galectin-3 Inhibits Osteoblast Differentiation through Notch Signaling. Neoplasia.

[34] Liu F., Malaval L., Gupta A.K., Aubin J.E. (1994). Simultaneous Detection of Multiple Bone-Related mRNAs and Protein Expression during Osteoblast Differentiation: Polymerase Chain Reaction and Immunocytochemical Studies at the Single Cell Level. Dev. Biol..

[35] Chen W.-G., Sun J., Shen W.-W., Yang S.-Z., Zhang Y., Hu X., Qiu H., Xu S.-C., Chu T.-W. (2019). Sema4D Expression and Secretion Are Increased by HIF-1α and Inhibit Osteogenesis in Bone Metastases of Lung Cancer. Clin. Exp. Metastasis.

[36] Mason J.B., Cargill S.L., Anderson G.B., Carey J.R. (2010). Ovarian Status Influenced the Rate of Body-Weight Change but Not the Total Amount of Body-Weight Gained or Lost in Female CBA/J Mice. Exp. Gerontol..

[37] Li S., Jiang H., Wang B., Gu M., Zhang N., Liang W., Wang Y. (2018). Effect of Leptin on Marrow Adiposity in Ovariectomized Rabbits Assessed by Proton Magnetic Resonance Spectroscopy. J. Comput. Assist. Tomogr..

[38] Beekman K.M., Veldhuis-Vlug A.G., den Heijer M., Maas M., Oleksik A.M., Tanck M.W., Ott S.M., van ’t Hof R.J., Lips P., Bisschop P.H. (2019). The Effect of Raloxifene on Bone Marrow Adipose Tissue and Bone Turnover in Postmenopausal Women with Osteoporosis. Bone.

[39] Wojdasiewicz P., Turczyn P., Lach-Gruba A., Poniatowski Ł.A., Purrahman D., Mahmoudian-Sani M.-R., Szukiewicz D. (2024). The Role of Rosavin in the Pathophysiology of Bone Metabolism. Int. J. Mol. Sci..

[40] Li D., Yuan T., Zhang X., Xiao Y., Wang R., Fan Y., Zhang X. (2012). Icariin: A Potential Promoting Compound for Cartilage Tissue Engineering. Osteoarthr. Cartil..

[41] Gao S., Cheng L., Li K., Liu W.-H., Xu M., Jiang W., Wei L.-C., Zhang F., Xiao W., Xiong Y. (2012). Effect of Epimedium Pubescen Flavonoid on Bone Mineral Status and Bone Turnover in Male Rats Chronically Exposed to Cigarette Smoke. BMC Musculoskelet. Disord..

[42] Wang J., Zhao B., Che J., Shang P. (2023). Hypoxia Pathway in Osteoporosis: Laboratory Data for Clinical Prospects. Int. J. Environ. Res. Public Health.

[43] Fujimoto H., Fujimoto K., Ueda A., Ohata M. (1999). Hypoxemia Is a Risk Factor for Bone Mass Loss. J. Bone Miner. Metab..

[44] Yang Y., Fan X., Tao J., Xu T., Zhang Y., Zhang W., Li L., Li X., Ding H., Sun M. (2018). Impact of Prenatal Hypoxia on Fetal Bone Growth and Osteoporosis in Ovariectomized Offspring Rats. Reprod. Toxicol..

[45] Fayed H.A., Barakat B.M., Elshaer S.S., Abdel-Naim A.B., Menze E.T. (2019). Antiosteoporotic Activities of Isoquercitrin in Ovariectomized Rats: Role of Inhibiting Hypoxia Inducible Factor-1 Alpha. Eur. J. Pharmacol..

[46] Knowles H.J. (2020). Distinct Roles for the Hypoxia-Inducible Transcription Factors HIF-1α and HIF-2α in Human Osteoclast Formation and Function. Sci. Rep..

[47] Wang X., Wei L., Li Q., Lai Y. (2022). HIF-1α Protects Osteoblasts from ROS-Induced Apoptosis. Free Radic. Res..

[48] Morita M., Sato Y., Iwasaki R., Kobayashi T., Watanabe R., Oike T., Miyamoto K., Toyama Y., Matsumoto M., Nakamura M. (2016). Selective Estrogen Receptor Modulators Suppress Hif1α Protein Accumulation in Mouse Osteoclasts. PLoS ONE.

[49] Chen D., Li Y., Zhou Z., Xing Y., Zhong Y., Zou X., Tian W., Zhang C. (2012). Synergistic Inhibition of Wnt Pathway by HIF-1α and Osteoblast-Specific Transcription Factor Osterix (Osx) in Osteoblasts. PLoS ONE.

[50] Li L., Li A., Zhu L., Gan L., Zuo L. (2022). Roxadustat Promotes Osteoblast Differentiation and Prevents Estrogen Deficiency-Induced Bone Loss by Stabilizing HIF-1α and Activating the Wnt/β-Catenin Signaling Pathway. J. Orthop. Surg. Res..

[51] Tang Y.-X., Liu M., Liu L., Zhen B.-R., Wang T.-T., Li N., Lv N., Zhu Z., Sun G., Wang X. (2022). Lipophilic Constituents in Salvia Miltiorrhiza Inhibit Activation of the Hepatic Stellate Cells by Suppressing the JAK1/STAT3 Signaling Pathway: A Network Pharmacology Study and Experimental Validation. Front. Pharmacol..

[52] Wang M., Zhao X., Zhu D., Liu T., Liang X., Liu F., Zhang Y., Dong X., Sun B. (2017). HIF-1α Promoted Vasculogenic Mimicry Formation in Hepatocellular Carcinoma through LOXL2 up-Regulation in Hypoxic Tumor Microenvironment. J. Exp. Clin. Cancer Res..

