# Supplementary material for: The Bioactive Compounds of Epimedium and Their Potential Mechanism of Action in Treating Osteoporosis: A Network Pharmacology and Experimental Validation Study
Source: Pharmaceuticals (Basel). 2024 May 29;17(6):706. doi: 10.3390/ph17060706 (PMC11206986; doi:10.3390/ph17060706)
Supplement: Supplementary file 1 [file pharmaceuticals-17-00706-s001.zip › pharmaceuticals-3004858-supplementary.pdf]

Supplementary Table S1. Database of Chemical Constituents of *Epimedium*

No.	Compound	Formula	Classification	No.	Compound	Formula	Classification
**1**	Linoleic Acid	C18H32O2	Acid	**59**	Icaritin-3-O-α-Rhamnoside	C27H32O11	Flavonoids
**2**	Succinic Acid	C4H6O4	Acid	**60**	Icariside E7	C29H38O13	Flavonoids
**3**	Stearin	C57H110O6	Acid	**61**	Icariside D3	C15H20O8	Flavonoids
**4**	p-Coumaric	C9H8O3	Acid	**62**	Icariside B9	C19H32O7	Flavonoids
**5**	Oleic Acid	C18H34O2	Acid	**63**	Icariside B6	C19H32O7	Flavonoids
**6**	5-p-Coumaroylquinic Acid	C16H18O8	Acid	**64**	Icariside A7	C23H26O10	Flavonoids
**7**	3-O-p-Coumaroylquinic Acid	C16H18O8	Acid	**65**	Icaride A2	C22H28O9	Flavonoids
**8**	Magnoflorine	C20H24NO4	Alkaloid	**66**	Hydnocarpin-D	C25H20O9	Flavonoids
**9**	Magnograndiolide	C15H22O4	Ester	**67**	(+/-)-Hydnocarpin	C25H20O9	Flavonoids
**10**	Linoleyl Acetate	C20H36O2	Ester	**68**	Hexandraside F	C39H52O19	Flavonoids
**11**	Hentriacontanol-6	C31H64O	Ester	**69**	Hexandraside D	C39H50O19	Flavonoids
**12**	Ceryl Alcohol	C26H54O	Ester	**70**	Epimedoside A	C32H38O15	Flavonoids
**13**	20-Hexadecanoylingenol	C36H58O6	Ester	**71**	Epimedokoreanoside II	C36H44O16	Flavonoids
**14**	Kaempferol	C15H10O6	Flavonoids	**72**	Epimedokoreanoside I	C44H56O22	Flavonoids
**15**	Icariside II	C27H32O10	Flavonoids	**73**	Epimedin A	C39H50O20	Flavonoids
**16**	Quercetin	C15H10O7	Flavonoids	**74**	Des-O-Methylicariin	C32H38O15223	Flavonoids
**17**	Icariin	C33H40O15	Flavonoids	**75**	Desmethylanhydroicaritin	C20H18O6	Flavonoids

**18**	Icarisid C	$C_{21}H_{38}O_{8}$	Flavonoids	**76**	Bilobanol	$C_{15}H_{22}O_{2}$	Flavonoids
**19**	Yinyanghuo C	$C_{20}H_{16}O_{5}$	Flavonoids	**77**	Baohuosu	$C_{22}H_{22}O_{7}$	Flavonoids
**20**	Epimedin B	$C_{38}H_{48}O_{19}$	Flavonoids	**78**	BaohuosideVII（Isomerism）	$C_{33}H_{40}O_{15}$	Flavonoids
**21**	Tricin	$C_{17}H_{14}O_{7}$	Flavonoids	**79**	Baohuoside VII	$C_{33}H_{40}O_{15}$	Flavonoids
**22**	Sagittatoside B	$C_{32}H_{38}O_{14}$	Flavonoids	**80**	Baohuoside HI	$C_{32}H_{40}O_{14}$	Flavonoids
**23**	Sagittatoside A	$C_{33}H_{40}O_{15}$	Flavonoids	**81**	Anhydroicaritin-3-O-α-L-Rhamnoside	$C_{27}H_{30}O_{10}$	Flavonoids
**24**	Kaempferitrin	$C_{27}H_{30}O_{14}$	Flavonoids	**82**	Acuminatoside	$C_{47}H_{64}O_{21}$	Flavonoids
**25**	Ikarisoside F	$C_{31}H_{36}O_{14}$	Flavonoids	**83**	6-Demethoxycapilarisin	$C_{15}H_{10}O_{6}$	Flavonoids
**26**	Icaritin	$C_{21}H_{20}O_{6}$	Flavonoids	**84**	5''-Methoxyhydnocarpin	$C_{26}H_{22}O_{10}$	Flavonoids
**27**	Icariside I	$C_{27}H_{30}O_{11}$	Flavonoids	**85**	5', 5''-Dimethoxyhydnocarpin-D	$C_{26}H_{22}O_{10}$	Flavonoids
**28**	Icarisid B	$C_{19}H_{32}O_{7}$	Flavonoids	**86**	Isomyricitrin	$C_{21}H_{20}O_{13}$	Flavonoids
**29**	SCHEMBL4214165	$C_{33}H_{40}O_{15}$	Flavonoids	**87**	Quercetin 3,7-Di-O-α-L-Rhamnopyranoside	$C_{27}H_{30}O_{15}$	Flavonoids
**30**	Hyperoside	$C_{21}H_{20}O_{12}$	Flavonoids	**88**	Hexandraside E	$C_{32}H_{38}O_{16}$	Flavonoids
**31**	Epimedin C	$C_{39}H_{50}O_{19}$	Flavonoids	**89**	Epimedin L	$C_{45}H_{56}O_{23}$	Flavonoids
**32**	Baohuoside II	$C_{26}H_{28}O_{10}$	Flavonoids	**90**	2’’-O-RhamnosylIcariside II	$C_{33}H_{40}O_{14}$	Flavonoids
**33**	Baohuoside I	$C_{27}H_{30}O_{10}$	Flavonoids	**91**	Diphylloside B	$C_{38}H_{48}O_{19}$	Flavonoids
**34**	Apigenin	$C_{15}H_{10}O_{5}$	Flavonoids	**92**	Yinyanghuo B	$C_{25}H_{26}O_{6}$	Flavonoids
**35**	Anhydroicaritin	$C_{45}H_{58}O$	Flavonoids	**93**	Yinyanghuo A	$C_{25}H_{24}O_{6}$	Flavonoids

		22					
**36**	7-O-Rhamnosyl icariside II	C33H42O14	Flavonoids	**94**	Yinyanghuo D	C20H18O5	Flavonoids
**37**	2"-Hydroxy-3"-En-Anhydroicaritin	C21H20O7	Flavonoids	**95**	3-[(2R,3S,4S,5S,6R)-4,5-dihydroxy-6-methyl-3-[(2R,3R,4S,5S,6R)-3,4,5-trihydroxy-6-(hydroxymethyl)oxan-2-yl]oxyoxan-2-yl]oxy-5,7-dihydroxy-2-(4-methoxyphenyl)-8-(3-methylbut-2-enyl)chromen-4-one	C33H40O15	Flavonoids
**38**	Icariside B2	C19H30O8	Flavonoids	**96**	Icarisid II	C27H32O10	Flavonoids
**39**	Icariside A1	C24H30O10	Flavonoids	**97**	3-[(6-Deoxy-alpha-L-mannopyranosyl)oxy]-7-(beta-D-glucopyranosyloxy)-5-hydroxy-2-(4-methoxyphenyl)-8-(3-methyl-2-buten-1-yl)-4H-1-benzopyran-4-one（SCHEMBL13038047）	C33H40O15	Flavonoids
**40**	Epimedoside C	C26H28O11	Flavonoids	**98**	Epimedin C（stereoisomerism）	C38H48O20	Flavonoids
**41**	Sagittatin B	C32H38O18	Flavonoids	**99**	Sagittatoside C	C35H42O16	Flavonoids
**42**	Baohuoside VI	C39H50O19	Flavonoids	**100**	Yixinoside A	C54H92O23	Glycosides
**43**	Yinyanghuo E	C20H16O6	Flavonoids	**101**	Diphylloside A	C36H44O20	Glycosides
**44**	Wushanicariin	C27H30O11	Flavonoids	**102**	(Z)-3-Hexenyl Glucoside	C12H22O6	Glycosides
**45**	Sagittatin A	C32H38O18	Flavonoids	**103**	Triacontane	C30H62	Hydrocarbons
**46**	Sagittasine B	C39H52O21	Flavonoids	**104**	Nonacosane	C29H60	Hydrocarbons

**47**	Sagittasine A	C39H52O21	Flavonoids	**105**	Hentriacontane	C31H64	Hydrocarbons
**48**	Rouhuoside	C38H48O20	Flavonoids	**106**	(+)-Cycloolivil	C20H24O7	Lignanoids
**49**	Quercetol	C30H26O15	Flavonoids	**107**	Wuweizi Alcohol B	C23H28O7	Lignanoids
**50**	Quercetin-3-O-a-L-Rhamnoside	C21H20O11	Flavonoids	**108**	Dihydrodehydrodiconiferyl Alcohol	C20H24O6	Lignanoids
**51**	Methoxyhydnocarpin-D	C25H20O9	Flavonoids	**109**	1,2-Bis(4-Hydroxy-3-Methoxyphenyl)Propan-1,3-Diol	C17H20O6	Others
**52**	Korepimedoside B	C46H60O22	Flavonoids	**110**	Chlorogenic	C16H18O9	Phenolic
**53**	Korepimedoside A	C36H44O17	Flavonoids	**111**	p-Hydroxybenzaldehyde	C7H6O2	Phenolic
**54**	Kaempferol-3-O-A-L-Rhamnoside	C21H20O10	Flavonoids	**112**	p-Hydroxyphenethyl	C8H10O2	Phenolic
**55**	Kaempferol-3-O-(3"-Z-P-Coumaroyl,4"-E-P-Coumaroyl)-A-L-Rhamnopyranoside	C37H32O12	Flavonoids	**113**	Blumenol C Glucoside	C19H32O7	Sesquiterpenes
**56**	Kaempferol-3-O-(2",4"-Di-Ep-Coumaroyl)-A-L-Rhamnopyranoside	C37H32O12	Flavonoids	**114**	Campesterol	C28H48O	Steroids
**57**	Ikarisoside C	C38H48O19	Flavonoids	**115**	β-Sitosterol	C29H50O	Steroids
**58**	Ikarisoside B	C32H38O15	Flavonoids	**116**	Inositol	C6H12O6	Vitamins

Supplementary Table S2. Known Anti-Osteoporosis Active Monomers from *Epimedium* and Their Mechanisms of Action

No.	Compound	Reported (*In vitro*)	Reported (*In vivo*)	In vivo/in vitro models	References
1	Chlorogenic	Promote proliferation of primary preosteoblasts in a dose-dependent manner, while attenuating alkaline phosphatase (ALP) activity.	Partially counteracts bone loss caused by ovariectomy by improving trabecular and cortical bone density, and increasing total bone mineral content; prevents the rise of bone formation marker osteocalcin and bone resorption marker urinary deoxypyridinoline caused by estrogen deficiency.	OVX	[16]
2	Kaempferol	Kaempferol prevents osteoporosis induced by OVX in rats by promoting osteoblast formation; increases the expression of alkaline phosphatase in BMSCs; upregulates the expression of Runx2 and Osterix; alters the phosphorylation of 4E/BP1 and S6K1; thereby promoting osteogenesis through the mTOR signaling pathway.	Treatment with Kaempferol significantly improved bone microarchitecture and bone quality in ovariectomized (OVX) rats.	OVX	[17]
2	Kaempferol	Kaempferol treatment increased the levels of BSP and Osterix mRNA, upregulated the expression of Runx2 mRNA, and enhanced the luciferase activity of the construct pLUC3; accelerated calcification in new bone formation areas. BSP antibody immunostaining revealed staining of the bone matrix and osteoblasts on the cranial surface. The thickness of the mineralized bone matrix stained with BSP antibody was more evident in the kaempferol group; osteoblast differentiation was in an activated state.	/	UMR106 cells	[18]
3	Icariside II	The osteogenic response of bone marrow mesenchymal stem cells to icariside II in vitro was evaluated using alkaline phosphatase (ALP) activity assay and Alizarin Red staining.	/	BMSCs	[19]
3	Icariside II	The osteogenic response of bone marrow mesenchymal stem cells to icariside II was assessed in vitro through alkaline phosphatase (ALP) activity assay and Alizarin Red staining. Western blot and RT-PCR were utilized to evaluate the expression of osteogenic proteins/genes. The impact of icariside II on the expression of osteogenic-related proteins/genes in bone marrow mesenchymal stem cells was examined.	/	BMSCs	[20]

4	Quercetin	/	The combined treatment of Quercetin and Vitamin E increased serum calcium levels, reduced serum osteocalcin levels, increased the number of osteoblasts and osteocytes, decreased the total number of osteoclasts, and increased overall trabecular volume, bone weight, and bone volume.	OVX	[21]
5	Icariin	Icariin prevents alveolar bone loss induced by estrogen deficiency by promoting osteogenesis through the STAT3 signaling pathway.	/	mBMSCs cells	[14]
6	Icarisid C	Affects UMR106 cell proliferation and alkaline phosphatase (ALP) activity.	/	UMR106 cells	[22]
7	Linoleic Acid	Linoleic Acid promotes osteogenesis in vitro; Linoleic Acid can inhibit the expression of PPAR γ in the femoral marrow cavity, suggesting its role in inhibiting adipose tissue generation in vitro.	Immunofluorescence of femoral sections indicates that Linoleic Acid promotes osteogenesis in vivo; Linoleic Acid can inhibit the expression of PPAR γ in the femoral marrow cavity, suggesting its role in inhibiting adipose tissue formation in vivo as well; it promotes bone formation in aged female C57/BL6 mice; it plays a significant role in osteogenesis and adipocyte differentiation through the mTORC1 signaling pathway.	MC3T3-E1 and 3T3-L1 cells; female C57/BL6 mice.	[23]
7	Linoleic Acid	/	Linoleic Acid significantly prevents the increase in body weight and fat mass associated with ovariectomy; significantly increases bone markers in the femur; significantly decreases femoral tartrate-resistant acid phosphatase (TRAP) activity, suggesting a potential decrease in osteoclastogenesis.	OVX	[24]
8	Magnoflorine	Treatment with Magnoflorine significantly increases the viability of MC3T3-E1 cells, enhances cell proliferation, and promotes mineralization. Moreover, it increases bone volume fraction, trabecular number, and connectivity density, while reducing anisotropy, indicating the regeneration of trabecular bone.	/	MC3T3-E1cells	[25]
9	Yinyanghuo C	/	Significantly increased bone weight and blood perfusion in BMP2-induced ectopic ossification mouse model, promoting bone maturation.	Ectopic ossification model	[26]

10	Epimedin B	/	Epimedin B significantly increases procollagen Type I N-terminal propeptide (P1NP) and decreases C-terminal telopeptide of Type I collagen (CTX1). After administration of Epimedin B, there is a significant increase in trabecular connectivity density and a significant decrease in anisotropy. Additionally, Epimedin B significantly increases the number of trabeculae and significantly reduces the gap between them. Through RNA sequencing, Epimedin B exerts a therapeutic effect on osteoporosis in mice by regulating the PI3K-Akt, MAPK, and PPAR signaling pathways.	Male ICR mice	[27]
11	Tricin	/	Tricin can promote the proliferation and mineralization of MSCs, enhance the expression of osteogenic marker genes such as osteopontin, osteocalcin, alkaline phosphatase, RUNX2, etc.; it can promote osteogenesis of human bone marrow mesenchymal stem cells through the regulation of the Wnt/β-catenin signaling pathway in vitro.	MSCs	[28]
12	Succinic Acid	In vitro, it can inhibit inflammation and osteoclastogenesis.	In vivo, it can reduce alveolar bone loss.	SUCNR1 gene knockout mice.	[29]
13	Stearin	Stearin increases the release of chondroitin sulfate proteoglycan from human chondrocytes.	Rats fed with Stearin showed improvements in cartilage degeneration and subchondral bone changes.	rats	[30]
14	Sagittatoside B	/	Treatment with icariin significantly improves bone strength in ovariectomized rats, markedly inhibits serum alkaline phosphatase and tartrate-resistant acid phosphatase levels. Compared to ovariectomized rats, icariin components also increase osteoprotegerin protein expression and decrease NF-κB ligand protein expression. μ-CT results demonstrate that icariin components significantly increase trabecular density in OVX	OVX	[31]

			rats, restoring bone microstructure. However, in-depth studies targeting individual components have not been conducted.		
15	Sagittatoside A	Promotes proliferation, alkaline phosphatase activity, and OPG/RANKL mRNA expression in UMR106 cells.	/	UMR106 Cells	[32]
16	p-Coumaric	p-Coumaric acid induces OPG production in osteoclast differentiation, maintaining calcium content.	The mannose-modified liposomal delivery system transports p-Coumaric to synovial macrophages in AIA rats, inhibiting TRAP staining, downregulating MMP-9 and NFATc1 expression, as well as inflammatory cytokines, thereby suppressing osteoclast differentiation.	AIA Rats	[33]
17	Kaempferitrin	/	Can significantly improve bone mass and microstructure in OVX rats, stimulate osteoblasts, inhibit osteoclasts, and downregulate the phosphorylation levels of I-κB.	OVX	[34]
18	Inositol	/	Continuous supplementation of inositol partially restores the abnormal bone phenotype in adult SMIT1 (-/-) mice and enhances bone structure in SMIT1 (+/+) mice.	C57BL/6/129Sv mice	[35]
19	Ikarisoside F	/	Treatment with icariin significantly improves bone strength in ovariectomized rats, markedly inhibits serum alkaline phosphatase and tartrate-resistant acid phosphatase levels. Compared to ovariectomized rats, icariin components also increase osteoprotegerin protein expression and decrease NF-κB ligand protein expression. μ-CT results demonstrate that icariin components significantly increase trabecular density in OVX rats, restoring bone microstructure. However, in-depth studies targeting individual components have not been conducted.	OVX	[31]
20	Icaritin	After treatment of BMSCs with Icaritin, their bone density decreased, alkaline phosphatase increased; formation of calcium nodules was observed; RUNX-2 and bFGF were significantly elevated; it can upregulate the phosphorylation level of MAPK/ERK.	/	BMSCs	[36]

21	Icariside I	/	Pharmacokinetics	Postmenopausal women	[37]
22	Icarisid B	Affects UMR106 cell proliferation and alkaline phosphatase (ALP) activity.	/	UMR106	[22]
23	SCHEMBL4214165 (Stereoisomerism of Icariin)	/	Lower blood glucose levels in diabetic rats and increase their bone density. Icariin effectively reduces serum bone turnover marker levels; bone histomorphometric parameters and weekly osteoclast counts return to normal levels, and icariin treatment inhibits bone marrow fat formation; the ratio of Runx2 to OPG/RANKL in serum and bone tissue of diabetic rats significantly increases.	Diabetic osteoporosis mice.	[38]
24	Hyperoside	/	Effectively prevents osteoporosis by increasing bone density, restoring the microstructure of trabeculae, and enhancing bone strength; significantly reduces the activity of bone resorption markers such as tartrate-resistant acid phosphatase 5b (TRAP-5b) and C-terminal telopeptide of type I collagen (CTX), and significantly increases the bioactivity of bone formation markers osteocalcin (OC) and bone-specific alkaline phosphatase (BALP); decreases the expression of receptor activator of nuclear factor-κB ligand (RANKL), TNF receptor-associated factor 6 (TRAF6), phospho-inhibitor of κBα (IκBα), NF-κB p65, and nuclear factor of activated T-cells cytoplasmic 1 (NFATc1), and promotes the expression of osteoprotegerin (OPG).	OVX	[39]
25	Epimedin C	Secondary osteoporosis is primarily caused by glucocorticoid (GC) treatment; enhances alkaline phosphatase activity and mineralization; stimulates the PI3K and AKT signaling pathways in dexamethasone-treated MC3T3-E1 cells.	Enhances bone mineralization in DEX-mediated bone injury; this effect may be reversed by PI3K inhibitors.	MC3T3-E1 cells and zebrafish larvae.	[40]
26	Campesterol	/	Consumption of beverages containing β-Cx and PS led to a significant increase in serum levels of β-Cx, β-sitosterol, and mannitol; only the intake of	Postmenopausal women	[41]

			beverages containing β-Cx and PS could significantly reduce total cholesterol, c-HDL, c-LDL, and bone turnover markers.		
27	β-sitosterol	There is significant proliferation of osteoblasts and alkaline phosphatase activity.	/	UMR-106	[42]
28	Baohuoside II	/	Significantly improved bone density in OVX rats and restored bone microarchitecture.	OVX	[31]
29	Baohuoside I	/	The anti-osteoporotic activity of Baohuoside I may be associated with inducing differentiation of bone marrow mesenchymal stem cells into osteoblasts, while inhibiting adipocyte formation, regulating immune function, and antioxidant activity.	OVX	[43]
30	Apigenin	At concentrations ranging from 1 to 10 μM, Apigenin significantly inhibits the proliferation of MC3T3-E1 osteoblast cells and osteoblast differentiation markers such as collagen production, alkaline phosphatase activity, and calcium deposition. At 10 μM, Apigenin completely inhibits the formation of multinucleated osteoclasts from mouse spleen cells.	Injecting Apigenin at a dose of 10 mg/kg body weight significantly inhibits trabecular bone loss in the femurs of OVX mice.	MC3T3-E1；OVX	[44]
31	Anhydroicaritin	Downregulates the levels of SREBP2 and its target genes in RANKL-induced osteoclasts without significant cytotoxicity; inhibits RANKL-induced osteoclast differentiation.	Lowers blood sugar levels, alleviates insulin resistance; inhibits osteoclast differentiation, rescues bone loss induced by diabetes.	RAW264.7; STZ (Streptozotocin)-induced diabetic mice.	[45]
32	7-O-RhamnosylIcariside II	/	Treatment with icariin significantly improves bone strength in ovariectomized rats, markedly inhibits serum alkaline phosphatase and tartrate-resistant acid phosphatase levels. Compared to ovariectomized rats, icariin components also increase osteoprotegerin protein expression and decrease NF-κB ligand protein expression. μ-CT results demonstrate that icariin components significantly increase trabecular density in OVX rats, restoring bone microstructure. However, in-depth studies targeting individual components	OVX	[31]

			have not been conducted.		
33	2"-Hydroxy-3"-en-anhydroicaritin	Enhanced proliferation of mouse osteoblastic MC3T3-E1 cells; increased alkaline phosphatase (ALP) activity levels in cells, significantly promoting the differentiation of MC3T3-E1 cells; Alizarin Red staining and quantification of mineralized nodules showed the potential to stimulate mineralized nodule formation and further accelerate bone formation.	/	MC3T3-E1	[46]
34	(+)-Cycloolivil	/	/	Through integrating active ingredient screening, drug similarity assessment, herb feature mapping, target prediction and validation, as well as network analysis, a systematic pharmacology was established to study the relationship between Epimedium and estrogen with osteoporosis.	[47]

35	Oleic Acid	/	In postmenopausal women with moderate cardiovascular risk, dietary supplementation with a milk-based beverage rich in fatty acids, oleic acid, minerals, and vitamins can have a positive effect on cardiovascular risk and bone metabolism parameters. Regular consumption may be a useful nutritional supplement for postmenopausal women.	Postmenopausal women	[48]
36	Icariside B2	Icariside B2 can reduce the expression of pro-inflammatory cytokines such as TNF-α, IL-6, and IL-1β at both transcriptional and translational levels; it inhibits the phosphorylation of IκBβ, thereby terminating the activation of NF-κB. Icariside B2 also inhibits the MAPK signaling pathway.	/	Mouse BV2 microglial cells.	[49]

Supplementary Table S3. Pairs of Potential Active Compounds with Tanimoto Similarity Greater Than 0.4(The No. was consistent with the compounds represented in **Supplementary Table S1**)

No.	Known active compounds	Potential active compounds	Similarity	No.	Known active compounds	Potential active compounds	Similarity	No.	Known active compounds	Potential active compounds	Similarity	No.	Known active compounds	Potential active compounds	Similarity
1	110	6	0.7368	184	21	93	0.4804	367	25	75	0.5156	550	98	95	0.7730
2	110	7	0.7368	185	21	94	0.5341	368	25	78	0.7279	551	98	96	0.5058
3	14	16	0.5733	186	3	103	0.4809	369	25	79	0.7047	552	98	97	0.8176
4	14	19	0.4222	187	3	104	0.4645	370	25	80	0.5696	553	98	98	0.8480
5	14	21	0.4091	188	3	11	0.4301	371	25	82	0.4785	554	98	99	0.6971
6	14	26	0.4043	189	3	105	0.4973	372	25	87	0.5096	555	114	115	0.8523
7	14	30	0.4369	190	22	23	0.8357	373	25	88	0.6178	556	32	33	0.8598
8	14	34	0.6765	191	22	24	0.5159	374	25	89	0.4737	557	32	35	0.4433

9	14	50	0.4216	192	22	25	0.8864	375	25	90	0.7552	558	32	36	0.4487
10	14	51	0.4128	193	22	26	0.5267	376	25	91	0.6450	559	32	37	0.4500
11	14	54	0.5714	194	22	27	0.5862	377	25	95	0.7517	560	32	40	0.7155
12	14	75	0.4828	195	22	17	0.7020	378	25	96	0.5556	561	32	41	0.4727
13	14	83	0.5811	196	22	30	0.4026	379	25	97	0.6282	562	32	42	0.5238
14	14	94	0.4598	197	22	98	0.6512	380	25	98	0.5876	563	32	44	0.4638
15	15	29	0.4872	198	22	32	0.6842	381	25	99	0.6688	564	32	45	0.4688
16	15	20	0.4278	199	22	33	0.7734	382	26	27	0.6330	565	32	46	0.4885
17	15	22	0.5442	200	22	35	0.5172	383	26	17	0.4539	566	32	47	0.4469
18	15	23	0.5467	201	22	36	0.5875	384	26	32	0.5780	567	32	48	0.5706
19	15	25	0.4737	202	22	37	0.4225	385	26	33	0.6509	568	32	50	0.6087
20	15	26	0.4344	203	22	40	0.5133	386	26	37	0.7442	569	32	54	0.6944
21	15	27	0.5074	204	22	41	0.5575	387	26	40	0.5487	570	32	55	0.4107
22	15	17	0.4872	205	22	42	0.6322	388	26	58	0.4532	571	32	56	0.4024
23	15	98	0.4309	206	22	45	0.5843	389	26	59	0.6509	572	32	57	0.5714
24	15	32	0.5308	207	22	46	0.5683	390	26	70	0.4028	573	32	58	0.7519
25	15	33	0.6290	208	22	47	0.5683	391	26	71	0.4792	574	32	59	0.8598
26	15	36	0.7891	209	22	48	0.5691	392	26	75	0.8125	575	32	68	0.5179
27	15	40	0.4225	210	22	50	0.4589	393	26	77	0.5455	576	32	69	0.5238
28	15	42	0.4309	211	22	53	0.4835	394	26	78	0.5074	577	32	70	0.6992
29	15	44	0.4041	212	22	54	0.4755	395	26	79	0.5074	578	32	71	0.6233
30	15	52	0.4819	213	22	57	0.6057	396	26	90	0.5188	579	32	72	0.4550
31	15	53	0.6781	214	22	58	0.7397	397	26	94	0.4592	580	32	73	0.5058
32	15	58	0.4774	215	22	59	0.7734	398	26	95	0.5074	581	32	101	0.5305
33	15	59	0.6290	216	22	68	0.6550	399	26	96	0.4957	582	32	74	0.4615
34	15	61	0.4248	217	22	69	0.6229	400	26	97	0.4539	583	32	75	0.6408
35	15	68	0.4333	218	22	70	0.6282	401	26	99	0.4792	584	32	77	0.4048
36	15	69	0.4309	219	22	71	0.7208	402	27	17	0.7279	585	32	78	0.6838
37	15	70	0.4313	220	22	72	0.5459	403	27	30	0.4923	586	32	79	0.6594
38	15	71	0.5000	221	22	73	0.6494	404	27	98	0.6074	587	32	80	0.5068
39	15	73	0.4239	222	22	101	0.5763	405	27	32	0.6423	588	32	82	0.4059
40	15	78	0.5467	223	22	74	0.4222	406	27	33	0.7227	589	32	86	0.4729
41	15	79	0.5263	224	22	75	0.4485	407	27	35	0.5294	590	32	87	0.4930
42	15	80	0.7121	225	22	78	0.8099	408	27	36	0.4321	591	32	88	0.6127
43	15	90	0.5578	226	22	79	0.7847	409	27	37	0.5126	592	32	89	0.4365
44	15	95	0.5467	227	22	80	0.5120	410	27	40	0.8636	593	32	90	0.6866

45	15	96	0.5075	228	22	82	0.5294	411	27	42	0.6074	594	32	91	0.5813
46	15	97	0.4872	229	22	87	0.4907	412	27	44	0.5639	595	32	95	0.6715
47	15	98	0.4309	230	22	88	0.5576	413	27	46	0.5143	596	32	96	0.6864
48	15	99	0.5190	231	22	89	0.5245	414	27	47	0.5143	597	32	97	0.6241
49	16	19	0.4130	232	22	90	0.8540	415	27	48	0.5503	598	32	98	0.5238
50	16	21	0.4483	233	22	91	0.6057	416	27	50	0.4254	599	32	99	0.6345
51	16	26	0.4255	234	22	95	0.8357	417	27	54	0.4651	600	33	35	0.4974
52	16	30	0.5000	235	22	96	0.6331	418	27	57	0.4971	601	33	36	0.5166
53	16	34	0.5065	236	22	97	0.7020	419	27	58	0.6000	602	33	37	0.5128
54	16	37	0.4316	237	22	98	0.6512	420	27	59	0.7227	603	33	40	0.6160
55	16	43	0.4348	238	22	99	0.7550	421	27	64	0.4444	604	33	41	0.4386
56	16	50	0.5978	239	23	24	0.5478	422	27	68	0.5263	605	33	42	0.5890
57	16	54	0.4500	240	23	25	0.7517	423	27	69	0.6074	606	33	44	0.4748
58	16	75	0.4886	241	23	26	0.5074	424	27	70	0.6691	607	33	45	0.4337
59	16	83	0.4512	242	23	27	0.6667	425	27	71	0.6419	608	33	46	0.5057
60	16	86	0.4757	243	23	17	0.8322	426	27	72	0.5440	609	33	47	0.5057
61	16	87	0.4032	244	23	30	0.4636	427	27	73	0.5774	610	33	48	0.5235
62	16	94	0.4828	245	23	98	0.7730	428	27	101	0.5118	611	33	50	0.5537
63	29	20	0.8165	246	23	32	0.6715	429	27	74	0.4096	612	33	53	0.4497
64	29	22	0.7020	247	23	33	0.7576	430	27	75	0.5351	613	33	54	0.5897
65	29	23	0.8322	248	23	35	0.6051	431	27	78	0.6549	614	33	57	0.5238
66	29	24	0.6093	249	23	36	0.6188	432	27	79	0.6786	615	33	58	0.6715
67	29	25	0.6282	250	23	37	0.4082	433	27	82	0.5104	616	33	61	0.4248
68	29	26	0.4539	251	23	40	0.5890	434	27	86	0.4519	617	33	68	0.5828
69	29	27	0.7279	252	23	41	0.5082	435	27	88	0.6434	618	33	69	0.5890
70	29	30	0.4539	253	23	42	0.7515	436	27	89	0.5211	619	33	70	0.6357
71	29	98	0.8176	254	23	44	0.4596	437	27	90	0.5890	620	33	71	0.7021
72	29	32	0.6241	255	23	45	0.5314	438	27	91	0.5602	621	33	72	0.5109
73	29	33	0.7059	256	23	46	0.6782	439	27	95	0.6667	622	33	73	0.5689
74	29	35	0.6738	257	23	47	0.6782	440	27	96	0.6016	623	33	101	0.4854
75	29	36	0.5235	258	23	48	0.6802	441	27	97	0.7279	624	33	74	0.4085
76	29	40	0.6454	259	23	50	0.4533	442	27	98	0.5322	625	33	75	0.5495
77	29	41	0.5249	260	23	52	0.4562	443	27	99	0.6200	626	33	77	0.4286
78	29	42	0.8291	261	23	53	0.5027	444	28	38	0.4688	627	33	78	0.7710
79	29	44	0.4688	262	23	54	0.4694	445	28	63	0.7250	628	33	79	0.7444
80	29	45	0.5141	263	23	57	0.6628	446	28	113	0.4681	629	33	80	0.4581

81	29	46	0.6977	264	23	58	0.8905	447	17	30	0.4539	630	33	82	0.4569
82	29	47	0.7076	265	23	59	0.7576	448	17	98	0.8176	631	33	86	0.4296
83	29	48	0.7410	266	23	68	0.7143	449	17	32	0.6241	632	33	87	0.4828
84	29	50	0.4437	267	23	69	0.7410	450	17	33	0.7059	633	33	88	0.5570
85	29	52	0.4562	268	23	70	0.7500	451	17	35	0.6738	634	33	89	0.4896
86	29	53	0.4474	269	23	71	0.8367	452	17	36	0.5235	635	33	90	0.7752
87	29	54	0.4694	270	23	72	0.6471	453	17	40	0.6454	636	33	91	0.5422
88	29	57	0.6628	271	23	73	0.7590	454	17	41	0.5249	637	33	95	0.7576
89	29	58	0.7383	272	23	101	0.5866	455	17	42	0.8291	638	33	96	0.8036
90	29	59	0.7059	273	23	74	0.5087	456	17	44	0.4688	639	33	97	0.7059
91	29	68	0.6744	274	23	75	0.4326	457	17	45	0.5141	640	33	98	0.5890
92	29	69	0.8291	275	23	78	0.9407	458	17	46	0.6977	641	33	99	0.7143
93	29	70	0.8905	276	23	79	0.9407	459	17	47	0.7076	642	34	43	0.5422
94	29	71	0.7419	277	23	80	0.5515	460	17	48	0.7410	643	34	51	0.5354
95	29	72	0.7207	278	23	82	0.6091	461	17	50	0.4437	644	34	54	0.4842
96	29	73	0.7697	279	23	86	0.4295	462	17	52	0.4562	645	34	66	0.4902
97	29	101	0.6416	280	23	87	0.5217	463	17	53	0.4474	646	34	67	0.4902
98	29	74	0.5174	281	23	88	0.6688	464	17	54	0.4694	647	34	75	0.4270
99	29	78	0.8322	282	23	89	0.6205	465	17	57	0.6628	648	34	83	0.7164
100	29	79	0.8322	283	23	90	0.8905	466	17	58	0.7383	649	34	85	0.4273
101	29	80	0.4798	284	23	91	0.7229	467	17	59	0.7059	650	34	92	0.4433
102	29	82	0.6256	285	23	96	0.6338	468	17	68	0.6744	651	34	93	0.4536
103	29	86	0.4204	286	23	97	0.8322	469	17	69	0.8291	652	34	94	0.6026
104	29	87	0.5705	287	23	98	0.6901	470	17	70	0.8905	653	35	36	0.4155
105	29	88	0.7945	288	23	99	0.8621	471	17	71	0.7419	654	35	40	0.4740
106	29	89	0.6720	289	4	111	0.5676	472	17	72	0.7207	655	35	41	0.4796
107	29	90	0.7383	290	4	6	0.4412	473	17	73	0.7697	656	35	42	0.6749
108	29	91	0.7546	291	4	7	0.4412	474	17	101	0.6416	657	35	45	0.4178
109	29	95	0.8322	292	24	25	0.5667	475	17	74	0.5174	658	35	46	0.5734
110	29	96	0.6000	293	24	27	0.4118	476	17	78	0.8322	659	35	47	0.5734
111	29	98	0.7305	294	24	17	0.6093	477	17	79	0.8322	660	35	48	0.6038
112	29	99	0.7419	295	24	30	0.4963	478	17	80	0.4798	661	35	52	0.5485
113	18	102	0.4045	296	24	98	0.5517	479	17	82	0.6256	662	35	53	0.4751
114	1	5	0.7538	297	24	32	0.5556	480	17	86	0.4204	663	35	57	0.5249
115	1	10	0.7143	298	24	33	0.5106	481	17	87	0.5705	664	35	58	0.5500
116	19	21	0.4945	299	24	35	0.4203	482	17	88	0.7945	665	35	59	0.4974

117	19	34	0.5750	300	24	36	0.4458	483	17	89	0.6720	666	35	68	0.5409
118	19	43	0.7317	301	24	40	0.4490	484	17	90	0.7383	667	35	69	0.6667
119	19	51	0.4435	302	24	41	0.7365	485	17	91	0.7546	668	35	70	0.6146
120	19	66	0.4821	303	24	42	0.5429	486	17	95	0.8322	669	35	71	0.6546
121	19	67	0.4821	304	24	45	0.7914	487	17	96	0.6000	670	35	72	0.8131
122	19	83	0.4494	305	24	46	0.5083	488	17	98	0.7305	671	35	73	0.6179
123	19	85	0.4370	306	24	47	0.5424	489	17	99	0.7419	672	35	101	0.5297
124	19	92	0.4128	307	24	48	0.5517	490	30	32	0.5285	673	35	78	0.5969
125	19	93	0.6316	308	24	50	0.5794	491	30	33	0.4806	674	35	79	0.6218
126	19	94	0.5618	309	24	54	0.6838	492	30	40	0.5403	675	35	82	0.5527
127	20	22	0.7516	310	24	55	0.4101	493	30	44	0.4809	676	35	87	0.4028
128	20	23	0.7716	311	24	56	0.4022	494	30	45	0.4188	677	35	88	0.5888
129	20	24	0.5056	312	24	57	0.5345	495	30	46	0.4101	678	35	89	0.7816
130	20	25	0.6807	313	24	58	0.5894	496	30	48	0.4011	679	35	90	0.5500
131	20	27	0.6149	314	24	59	0.5106	497	30	50	0.7525	680	35	91	0.5972
132	20	17	0.8165	315	24	68	0.5028	498	30	54	0.6667	681	35	95	0.6051
133	20	98	0.8802	316	24	69	0.5517	499	30	58	0.4932	682	35	96	0.4293
134	20	32	0.5301	317	24	70	0.6667	500	30	59	0.4806	683	35	97	0.6738
135	20	33	0.5963	318	24	71	0.4852	501	30	64	0.4365	684	35	98	0.6038
136	20	35	0.6488	319	24	72	0.4523	502	30	70	0.4830	685	35	99	0.6294
137	20	36	0.5106	320	24	73	0.4837	503	30	71	0.4494	686	36	41	0.4219
138	20	40	0.5482	321	24	101	0.5143	504	30	74	0.4013	687	36	42	0.5376
139	20	41	0.6183	322	24	74	0.5125	505	30	78	0.4539	688	36	45	0.4481
140	20	42	0.8580	323	24	78	0.5677	506	30	79	0.4733	689	36	46	0.5052
141	20	44	0.4054	324	24	79	0.5478	507	30	86	0.7670	690	36	47	0.4821
142	20	45	0.6648	325	24	80	0.4813	508	30	87	0.5455	691	36	48	0.4518
143	20	46	0.7611	326	24	86	0.4571	509	30	88	0.4733	692	36	52	0.5729
144	20	47	0.7709	327	24	87	0.8374	510	30	90	0.4065	693	36	53	0.7325
145	20	48	0.7640	328	24	88	0.5987	511	30	91	0.4080	694	36	57	0.4663
146	20	52	0.4698	329	24	89	0.4211	512	30	95	0.4636	695	36	58	0.5422
147	20	53	0.4423	330	24	90	0.6000	513	30	96	0.4806	696	36	59	0.5166
148	20	54	0.4012	331	24	91	0.5988	514	30	97	0.4539	697	36	68	0.5489
149	20	57	0.7088	332	24	95	0.5478	515	30	99	0.4313	698	36	69	0.5376
150	20	58	0.7006	333	24	96	0.5106	516	98	32	0.5238	699	36	70	0.4629
151	20	59	0.5963	334	24	97	0.6093	517	98	33	0.5890	700	36	71	0.5434
152	20	68	0.6649	335	24	98	0.5084	518	98	35	0.6585	701	36	72	0.4455

153	20	69	0.8471	336	24	99	0.4940	519	98	36	0.5628	702	36	73	0.5052
154	20	70	0.7423	337	25	26	0.4701	520	98	40	0.5417	703	36	101	0.4121
155	20	71	0.6761	338	25	27	0.5235	521	98	41	0.5619	704	36	74	0.4098
156	20	72	0.7254	339	25	17	0.6282	522	98	42	0.9506	705	36	78	0.6087
157	20	73	0.8218	340	25	30	0.4295	523	98	44	0.4011	706	36	79	0.5890
158	20	101	0.7167	341	25	98	0.5876	524	98	45	0.6209	707	36	80	0.8333
159	20	74	0.4667	342	25	32	0.7680	525	98	46	0.8655	708	36	82	0.4673
160	20	78	0.7500	343	25	33	0.6842	526	98	47	0.8655	709	36	87	0.4320
161	20	79	0.7500	344	25	35	0.4663	527	98	48	0.8057	710	36	88	0.4076
162	20	80	0.4635	345	25	36	0.5120	528	98	52	0.4784	711	36	89	0.4292
163	20	82	0.6847	346	25	40	0.5775	529	98	53	0.4519	712	36	90	0.6954
164	20	87	0.4835	347	25	41	0.5952	530	98	57	0.7293	713	36	91	0.5134
165	20	88	0.6784	348	25	42	0.5698	531	98	58	0.7024	714	36	95	0.6188
166	20	89	0.6634	349	25	45	0.6250	532	98	59	0.5890	715	36	96	0.4224
167	20	90	0.6905	350	25	46	0.5519	533	98	68	0.7213	716	36	97	0.5235
168	20	91	0.8081	351	25	47	0.5106	534	98	69	0.9506	717	36	98	0.5213
169	20	95	0.7716	352	25	48	0.6149	535	98	70	0.7439	718	36	99	0.5614
170	20	96	0.5118	353	25	50	0.4894	536	98	71	0.6875	719	37	40	0.4508
171	20	97	0.8165	354	25	53	0.4202	537	98	72	0.7632	720	37	59	0.5128
172	20	98	0.7943	355	25	54	0.5407	538	98	73	0.8439	721	37	75	0.6333
173	20	99	0.6857	356	25	57	0.6548	539	98	101	0.6720	722	37	78	0.4082
174	21	34	0.5443	357	25	58	0.8321	540	98	74	0.4619	723	37	79	0.4082
175	21	43	0.5682	358	25	59	0.6842	541	98	78	0.7515	724	37	90	0.4167
176	21	51	0.5140	359	25	68	0.5909	542	98	79	0.7515	725	37	92	0.4035
177	21	66	0.4862	360	25	69	0.5611	543	98	80	0.4895	726	37	95	0.4082
178	21	67	0.4862	361	25	70	0.6959	544	98	82	0.7286	727	37	96	0.5000
179	21	77	0.6292	362	25	71	0.6375	545	98	87	0.5281	728	106	108	0.4848
180	21	83	0.4535	363	25	72	0.4925	546	98	88	0.6802	729	106	109	0.4271
181	21	84	0.4153	364	25	73	0.5866	547	98	89	0.6897	730	5	10	0.6000
182	21	85	0.5755	365	25	101	0.6235	548	98	90	0.7546	731	38	63	0.5161
183	21	92	0.5152	366	25	74	0.4795	549	98	91	0.9085				

Supplementary Table S4. Potential Target Proteins for *Epimedium* Against Osteoporosis

Uniprot ID	Count	Classification	Uniprot ID	Count	Classification	Uniprot ID	Count	Classification	Uniprot ID	Count	Classification
O76074	48	Enzyme	P07451	8	Enzyme	O14684	6	Enzyme	O60285	4	Kinase
P15121	34	Enzyme	P05164	8	Enzyme	B2RXH2	6	Enzyme	O00748	4	Enzyme
P00918	34	Enzyme	P05067	8	Other	Q9HC97	5	G-Protein Coupled Receptor	Q9Y2D0	3	Enzyme
O43570	29	Enzyme	P03956	8	Peptidase	Q96S37	5	Transporter	Q9NWZ3	3	Kinase
P22748	28	Enzyme	Q9Y233	7	Enzyme	CDK2	5	Kinase	Q9HCG7	3	Enzyme
P22303	27	Enzyme	Q9UM73	7	Kinase	P78396	5	Other	Q92630	3	Kinase
Q9NPH5	24	Enzyme	Q9UHC9	7	Transporter	P20248	5	Other	Q8WWX8	3	Transporter
P43166	24	Enzyme	Q9HC98	7	Kinase	Q16875	5	Kinase	Q16739	3	Enzyme

P35354	20	Enzyme	Q8N1Q1	7	Enzyme	Q16831	5	Enzyme	Q15722	3	G-Protein Coupled Receptor
P30542	19	G-Protein Coupled Receptor	Q16539	7	Kinase	Q16512	5	Kinase	Q15465	3	Peptidase
P08183	19	Transporter	Q13332	7	Phosphatase	Q14994	5	Ligand-Dependent Nuclear Receptor	Q14680	3	Kinase
P51812	18	Kinase	Q04828	7	Enzyme	Q13554	5	Kinase	Q13564	3	Enzyme
P28907	18	Enzyme	Q02750	7	Kinase	Q13133	5	Ligand-Dependent Nuclear Receptor	Q07817	3	Other
P11511	18	Enzyme	Q02156	7	Kinase	Q08499	5	Enzyme	P63316	3	Other
P47989	17	Enzyme	P80365	7	Enzyme	Q07820	5	Transporter	P49810	3	Peptidase
P16083	17	Enzyme	P53355	7	Kinase	Q05397	5	Kinase	P49354	3	Enzyme
P00533	16	Kinase	P53350	7	Kinase	Q04760	5	Enzyme	P52333	3	Kinase
Q9GZQ4	15	G-Protein Coupled Receptor	P51955	7	Kinase	P51449	5	Ligand-Dependent Nuclear Receptor	P48147	3	Peptidase
P45452	15	Peptidase	P47871	7	G-Protein Coupled Receptor	P35228	5	Enzyme	P43116	3	G-Protein Coupled Receptor
P18825	15	G-Protein Coupled Receptor	P36888	7	Kinase	P30304	5	Phosphatase	P42345	3	Kinase
P09917	15	Enzyme	P35869	7	Ligand-Dependent Nuclear Receptor	P24723	5	Kinase	P42336	3	Kinase
P08913	15	G-Protein Coupled Receptor	P35218	7	Enzyme	P23219	5	Enzyme	P34995	3	G-Protein Coupled Receptor
Q92731	14	Ligand-Dependent Nuclear Receptor	P31639	7	Transporter	P13569	5	Ion Channel	P29350	3	Phosphatase

P29274	14	G-Protein Coupled Receptor	P27986	7	Kinase	P11142	5	Enzyme	P29275	3	G-Protein Coupled Receptor
P18031	14	Phosphatase	P24941	7	Kinase	P11021	5	Enzyme	P25025	3	G-Protein Coupled Receptor
P17252	14	Kinase	P23280	7	Enzyme	P09467	5	Phosphatase	P23975	3	Transporter
P06737	13	Enzyme	P22894	7	Peptidase	P07550	5	G-Protein Coupled Receptor	P23946	3	Peptidase
P03372	13	Ligand-Dependent Nuclear Receptor	P21917	7	G-Protein Coupled Receptor	P06746	5	Enzyme	P21554	3	G-Protein Coupled Receptor
P60568	12	Cytokine	P14780	7	Peptidase	P05093	5	Enzyme	P20292	3	Enzyme
P56817	12	Peptidase	P14061	7	Enzyme	P04062	5	Enzyme	P20151	3	Peptidase
P39900	12	Peptidase	P12931	7	Kinase	Q9NY91	4	Transporter	P17948	3	Kinase
P10275	12	Ligand-Dependent Nuclear Receptor	P11474	7	Transcription Regulator	Q9GZT9	4	Enzyme	P17936	3	Other
O60218	12	Enzyme	P09874	7	Enzyme	Q96RI1	4	Ligand-Dependent Nuclear Receptor	P17706	3	Phosphatase
Q99808	11	Transporter	P08254	7	Peptidase	Q969F8	4	G-Protein Coupled Receptor	P15090	3	Transporter
Q16678	11	Enzyme	P08238	7	Enzyme	Q8TDU6	4	G-Protein Coupled Receptor	P15056	3	Kinase
P33527	11	Transporter	P07900	7	Enzyme	Q7Z2W7	4	Ion Channel	P14625	3	Other
P11387	11	Enzyme	P06276	7	Enzyme	Q16850	4	Enzyme	P14416	3	G-Protein Coupled Receptor
P0DMS8	11	G-Protein Coupled Receptor	P04150	7	Ligand-Dependent Nuclear Receptor	Q15858	4	Ion Channel	P11413	3	Enzyme

P08253	11	Peptidase	O96017	7	Kinase	Q15746	4	Kinase	P09884	3	Enzyme
P00734	11	Peptidase	O95271	7	Enzyme	Q15172	4	Phosphatase	P08908	3	G-Protein Coupled Receptor
Q9UNQ0	10	Transporter	O43868	7	Transporter	Q15118	4	Kinase	P08311	3	Peptidase
P68400	10	Kinase	O14757	7	Kinase	Q13627	4	Kinase	P08235	3	Ligand-Dependent Nuclear Receptor
P06493	10	Kinase	Q9H2K2	6	Enzyme	Q05655	4	Kinase	P08185	3	Other
P04278	10	Other	Q96GD4	6	Kinase	Q03181	4	Ligand-Dependent Nuclear Receptor	P08151	3	Transcription Regulator
P01375	10	Cytokine	Q00534	6	Kinase	P78527	4	Kinase	P06870	3	Peptidase
P00915	10	Enzyme	P55263	6	Kinase	P53779	4	Kinase	P06239	3	Kinase
O14746	10	Enzyme	P52895	6	Enzyme	P52789	4	Kinase	P05413	3	Transporter
Q16790	9	Enzyme	P40763	6	Transcription Regulator	P50570	4	Enzyme	P05186	3	Phosphatase
Q15078	9	Kinase	P30530	6	Kinase	P48736	4	Kinase	P05121	3	Other
P37231	9	Ligand-Dependent Nuclear Receptor	P30518	6	G-Protein Coupled Receptor	P36897	4	Kinase	P04745	3	Enzyme
P35968	9	Kinase	P30305	6	Phosphatase	P35398	4	Ligand-Dependent Nuclear Receptor	O75688	3	Phosphatase
P31749	9	Kinase	P29372	6	Enzyme	P31645	4	Transporter	O60674	3	Kinase
P28845	9	Enzyme	P25024	6	G-Protein Coupled Receptor	P30556	4	G-Protein Coupled Receptor	O00754	3	Enzyme
P18054	9	Enzyme	P21397	6	Enzyme	P28482	4	Kinase	O00519	3	Enzyme
P16050	9	Enzyme	P17516	6	Enzyme	P28221	4	G-Protein Coupled Receptor	P45379	3	Other
P14679	9	Enzyme	P13866	6	Transporter	P27695	4	Enzyme	P19429	3	Transporter
P04054	9	Enzyme	P11388	6	Enzyme	P19367	4	Kinase	Q9NZ42	3	Peptidase
Q00535	9	Kinase	P10636	6	Other	P16662	4	Enzyme	Q92542	3	Peptidase

Q9ULX7	8	Enzyme	P08588	6	G-Protein Coupled Receptor	P14550	4	Enzyme	Q96BI3	3	Peptidase
P49841	8	Kinase	P08581	6	Kinase	P10721	4	Transmembrane Receptor	P49768	3	Peptidase
P43405	8	Kinase	P08069	6	Transmembrane Receptor	P06401	4	Ligand-Dependent Nuclear Receptor	Q16665	3	Other
P42330	8	Enzyme	P05771	6	Kinase	P04626	4	Kinase	Q8WW43	3	Peptidase
P37059	8	Enzyme	P05089	6	Enzyme	P04066	4	Enzyme	P49356	3	Enzyme
P11309	8	Kinase	P04035	6	Enzyme	P00742	4	Peptidase			
P09237	8	Enzyme	P02766	6	Enzyme	P00491	4	Enzyme			

Supplementary Table S5. Potential target proteins in HIF-1α Pathway

Molecule	Known/unknown target for osteoporosis (✔/×)	References	Molecule	Known/unknown target for osteoporosis (✔/×)	References
AKT1	✔	[26]	MMP2	×	
APEX1	×		MMP3	✔	[27]
BRAF	×		MMP7	×	
CAMK2B	×		MMP8	×	
EGLN1	×		MMP9	✔	[28]
FLT1	✔	[29]	MTOR	✔	[30]
HIF1A	✔	[31]	NOS2	×	
HK1	×		NOX4	✔	[32]
HK2	✔	[33]	PIK3CA	✔	[34]
HSP90AA1	×		PIK3CG	×	
HSPA5	✔	[35]	PIK3R1	✔	[36]
HSPA8	×		PRKCA	×	
KDR	×		PRKCB	×	
MAP2K1	×		PRKCD	×	
MAPK1	×		PRKCE	×	
MET	×		PRKCH	×	
MMP1	×		RORC	×	
MMP12	×		SERPINE1	×	
MMP13	✔	[37]	STAT3	✔	[38]

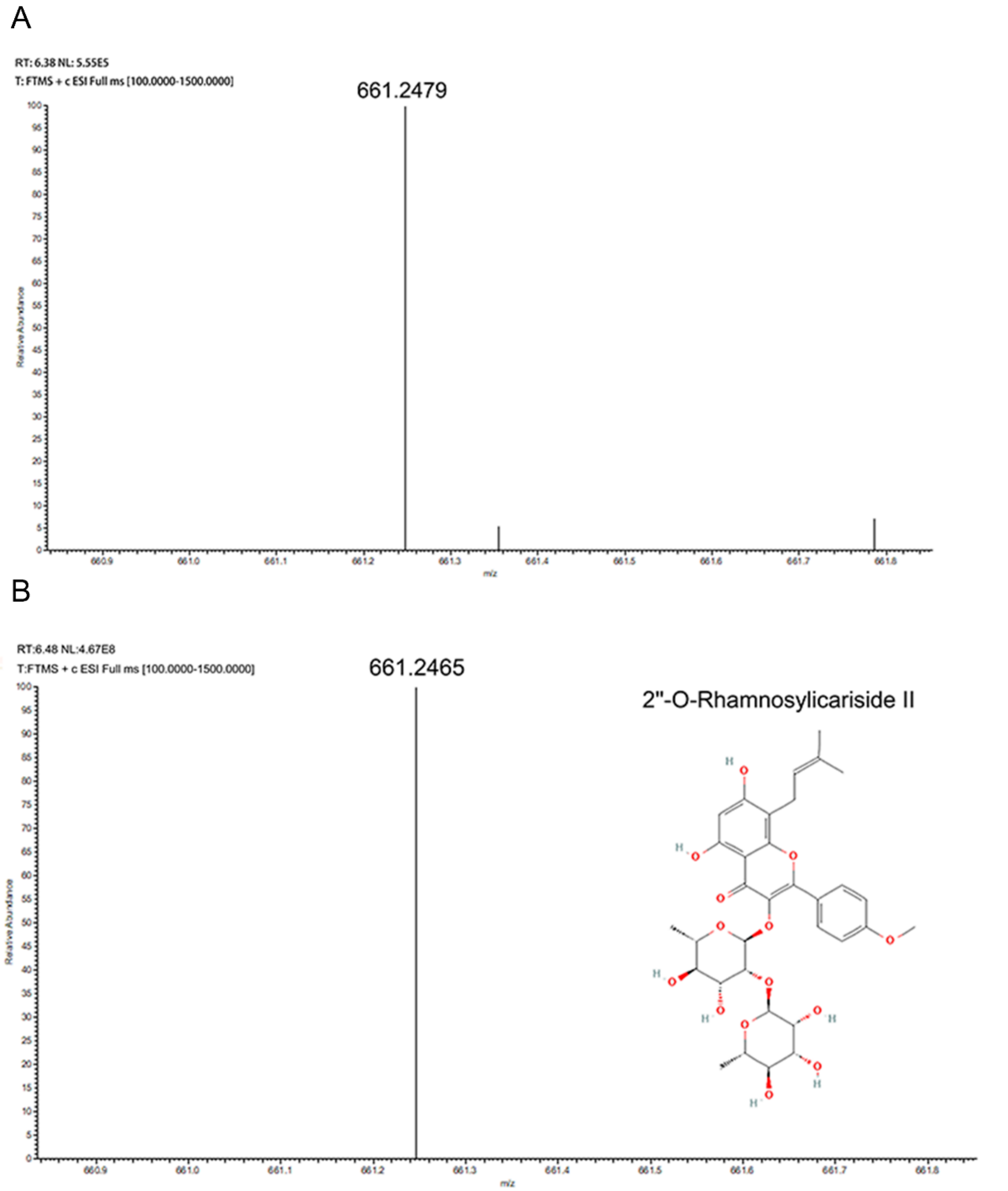

Supplementary figure S1. The presence of 2"-O-RhamnosylIcariside II in the aqueous *Epimedium* extract. **(A)** Positive ion mode MS spectrum of the *Epimedium* aqueous extract. **(B)** Positive ion mode MS spectrum of the 2"-O-RhamnosylIcariside II standard.

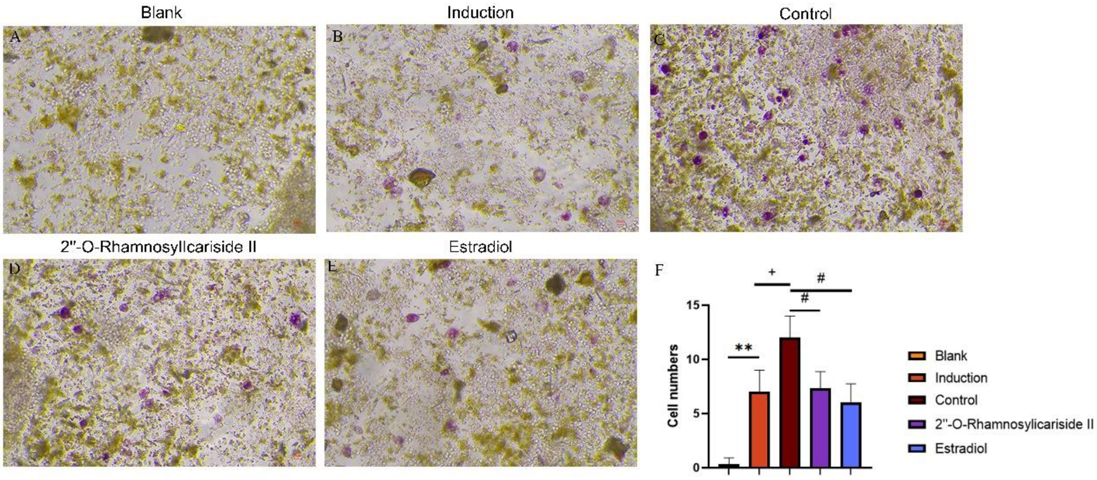

Supplementary figure S2. TRAP staining analysis of the effect of 2''-O-rhamnosylicariside II on the ability of RAW264.7 cells to differentiate into osteoclasts. The osteoclast induction medium consisted of HDMEM, 10% fetal bovine serum, 1% penicillin/streptomycin, and RANKL (50 ng/mL). The concentration of 2''-O-rhamnosylicariside II for RAW264.7 was 50μM.(A) Blank group. The cells were cultured in HDMEM. (B) Induction group. The cells were cultured in osteoclast induction medium. (C) Control group. The cells were cultured in osteoclast induction medium supplemented with $CoCl_2$ (100 μM) on the 4th day. (D) 2''-O-rhamnosylicariside II group. The cells were cultured in osteoclast induction medium supplemented with 2''-O-rhamnosylIcariside II (50 μM) and further treated with $CoCl_2$ (100 μM) on the 4th day. (E) Estradiol group. The cells were cultured in osteoclast induction medium supplemented with estradiol (100 μM) added and further treated with $CoCl_2$ (100 μM) on the 4th day. (F) Number of osteoclasts in each group as showed by TRAP staining. The data are presented as the mean ± standard error of the mean (SEM) (n = 3); $^{**}p<0.01$ indicates a statistically significant difference compared to the blank group, $^{+}p<0.05$ indicates a statistically significant difference compared to the control group, and $^{\#}p<0.05$ indicates a statistically significant difference compared to the control + $CoCl_2$ group.
